# A cucurbit[8]uril-triggered ionic photosensitizer in solution and solid states: selective control of ^1^O_2_ and O_2_˙^−^ generation

**DOI:** 10.1039/d5sc06904a

**Published:** 2026-01-06

**Authors:** Haigen Nie, Jiao Tan, Yi Luo, Xin-long Ni

**Affiliations:** a Key Laboratory of Chemical Biology and Traditional Chinese Medicine, Ministry of Education of China, Key Laboratory of the Assembly and Application of Organic Functional Molecules of Hunan Province, Hunan Normal University Changsha 410081 China longni333@163.com

## Abstract

Selective control of reactive oxygen species (ROS) generation captures the imagination of scientists because of its broad potential applications in photochemical reactions and biomedicine. Herein, we develop a novel supramolecular method enabling selective control of ^1^O_2_ and O_2_˙^−^ generation based on host–guest assembly in solution and the solid state. The cationic guest G-I (Cl^−^ as counteranions) lacks the ability to sensitize ROS but is transformed into an efficient organic photosensitizer through face-to-face dimerization within the cucurbit[8]uril (Q[8] or CB[8]) cavity *via* host–guest interactions. Although the G-I@Q[8] complex retains an identical assembly structure in both solution and solid-state phases, the differing electron transfer pathways of Cl^−^ counteranions between phases result in selective control of ^1^O_2_ and O_2_˙^−^ generation. This control is readily achievable by employing the host–guest complex as homogeneous or heterogeneous photocatalysts. Importantly, X-ray structural analysis reveals that the dimerized G-I@Q[8] framework exhibits remarkable formaldehyde (HCHO) adsorption capability due to the outer-surface interactions of the Q[8] host, enabling the solid G-I@Q[8] complex to serve as a highly efficient adsorption–photocatalytic platform for HCHO remediation. This study advances our understanding of macrocycle-mediated host–guest assembly in controlling ROS generation and photocatalysts with multiple functions.

## Abstract 2

A supramolecular approach selectively generates ^1^O_2_/O_2_˙^−^*via* host–guest assembly. The dimeric G-I@Q[8] framework adsorbs formaldehyde (HCHO), which is then photocatalytically degraded to formic acid (HCOOH) under blue light or sunlight.

## Graphical abstract

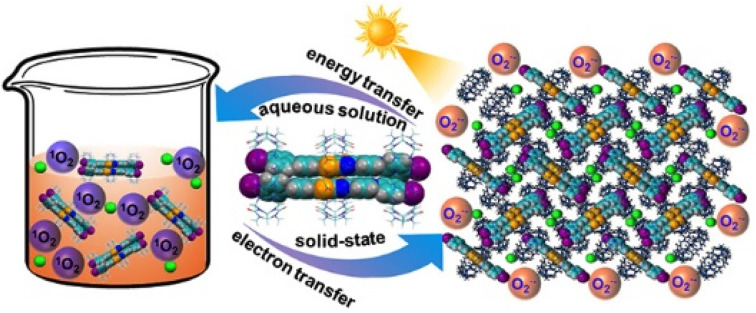

## Introduction

Singlet oxygen (^1^O_2_) and superoxide radicals (O_2_˙^−^) are the two most common reactive oxygen species (ROS).^1,2^ They serve as environmentally friendly oxidants in photochemical reactions and biomedicine. For example, ^1^O_2_ displays remarkable electrophilicity and selectivity in the oxidation of electron rich substrates,^3,4^ while O_2_˙^−^ is implicated as a reactive intermediate in many chemical mechanisms, exhibiting multiple reaction pathways.^5–7^ However, ROS generation often involves competitive production between ^1^O_2_ and O_2_˙^−^, which leads to elusive reaction pathways and reduced selectivity of catalytic products.^1,8,9^ Hence, the revelation of ROS generation mechanisms and establishment of selective control strategies for ^1^O_2_ and O_2_˙^−^ production represent an urgent but formidable challenge.^10–12^

In principle, ^1^O_2_ is produced *via* energy transfer between spin-matched triplet excitons and ground state oxygen (O_2_), whereas O_2_˙^−^ is generated *via* electron transfer from photosensitizers (PSs) to O_2_.^13^ In these processes, PSs play a key role in light absorption and the transfer of energy and electrons, thereby regulating the formation of ROS. Traditional PSs such as metal complexes,^14^ BODIPY,^15^ porphyrin and their derivatives^16^ exhibited excellent ability to sensitize O_2_ to produce ROS. However, the elusive selectivity in generating ROS and substantial photobleaching significantly inhibited their potential application. In this regard, numerous efforts have been made to identify the critical photosensitizing factors (*e.g.* excited state type, lifetime, redox potential, and energy level) that influence ROS production.^12,17–19^ For example, Yang *et al.* incorporated a series of BODIPY units at the α,β-positions to lower the reduction potential of the T_1_ state, resulting in an energy gap of T_1_–S_0_ smaller than that between ^3^O_2_ and ^1^O_2_, which enhanced the efficiency of O_2_˙^−^ generation.^17^ Jiang and co-workers demonstrated that selective ^1^O_2_/O_2_˙^−^ generation could be achieved by manipulating the formation and dissociation of triplet excitons in porphyrinic COFs by introducing Zn^2+^ and Ni^2+^, respectively.^18^ Nevertheless, developing materials with ordered molecular structures to systematically regulate photosensitizing factors and ROS generation remains highly challenging, as it requires precise molecular-level fabrication/modification and entails tedious, multi-step organic synthesis.

Supramolecular assembly has recently emerged as a facile, low cost method for fabricating efficient photosensitizers (PSs) and photocatalysts.^20–22^ In this approach, well-ordered architectures capable of efficient ROS generation spontaneously form from individual PS components through noncovalent interactions or self-aggregation.^23–25^ Among these strategies, macrocycle-based host–guest interactions exhibit distinct advantages for activating ROS generators, owing to the macrocycle's ability to precisely regulate the electronic distribution of guests within its cavity.^26–31^ For instance, Q[8], a prominent member of the cucurbit[*n*]uril ([Q[*n*] or CB[*n*])^32,33^ family, demonstrates unique properties distinct from other macrocycles. These properties stem primarily from its capacity to stabilize ternary complexes by encapsulating two hetero- or homogeneous guests within its cavity in aqueous media with high binding constants.^34–44^ Recent studies reveal that the rigid macrocyclic confinement of Q[8] can efficiently facilitate intersystem crossing (ISC) of the encapsulated guests, promoting triplet exciton formation and thereby enhancing ROS generation efficiency.^45–52^ Notably, the Q[8]-triggered host–guest interaction converts ROS-incapable guest molecules into efficient PSs. This provides a novel strategy for preparing efficient photocatalysts, offering both low cost (by avoiding complex organic synthesis and noble metals) and simple operation (achieved simply by adding the host to guest molecule solutions to form host–guest complexes).

**Scheme 1:**
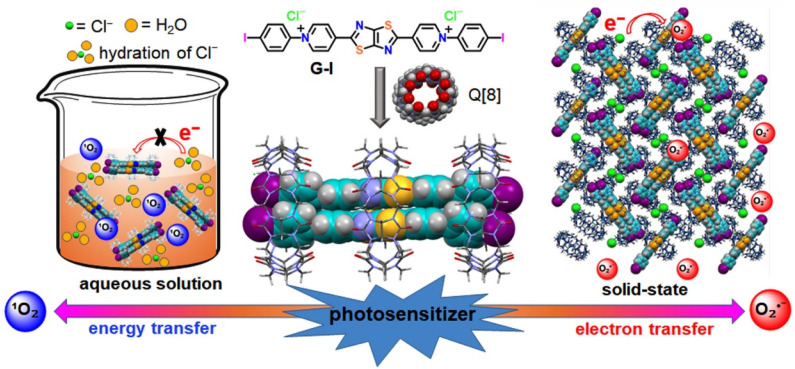
Chemical structures and schematic representation of Q[8]-based supramolecular assemblies and selective control of ^1^O_2_ and O_2_˙^−^ generation.

Herein, a novel Q[8]-based host–guest assembly constructed ionic PS (G-I@Q[8]) was first exploited to selectively control ^1^O_2_ and O_2_˙^−^ generation. As illustrated in Scheme 1, free cationic G-I molecules lack the ability to generate reactive oxygen species (ROS); however, they are transformed into effective ROS sensitizers upon complexation with the Q[8] host. This enhancement arises from macrocyclic confinement triggered dimerization of G-I in a perfect face-to-face configuration within the cavity, which promotes intersystem crossing (ISC) and spin–orbit coupling (SOC). Interestingly, although the G-I@Q[8] complex retains an identical assembly structure in both solution and solid-state phases, the differing electron transfer pathways of Cl^−^ counteranions between phases result in selective control of ^1^O_2_ and O_2_˙^−^ generation. This control is readily achievable by employing the host–guest complex as homogeneous or heterogeneous photocatalysts. Furthermore, single-crystal X-ray structural analysis revealed that the dimerized G-I@Q[8] frameworks strongly adsorb formaldehyde (HCHO) *via* outer-surface interactions^53,54^ with the Q[8] host in the solid state. This dual functionality—adsorption and photocatalytic degradation—enables the G-I@Q[8] complex to serve as an efficient platform for HCHO capture and degradation. This work highlights the novel application of Q[*n*]-mediated supramolecular assemblies, leveraging both cavity confinement and outer-surface interactions, in advancing functional materials science.

## Results and discussion

We recently discovered that thiazolo[5,4-*d*]thiazole *N*,*N*′-diarylpyridine (TTDP) derivatives—featuring a donor–acceptor–donor (D–A–D) structure—act as guests capable of forming unique 3 : 2 (host/guest) assemblies with Q[8] in aqueous solution.^52^ Within the Q[8] cavity, these guests dimerize in a face-to-face configuration. The macrocycle-confined dimeric complex exhibits an efficient intersystem crossing (ISC) process and demonstrates wavelength-dependent selective photooxidation activity in water. These findings prompted us to investigate the heavy-atom effect in this host–guest system, as introducing heavy atoms (*e.g.*, iodine) can further enhance ISC efficiency by increasing spin–orbit coupling (SOC). Accordingly, an iodine-substituted thiazolo[5,4-*d*]thiazole *N*,*N*′-diarylpyridine derivative was synthesized (G-I; SI).

**Fig. 1:**
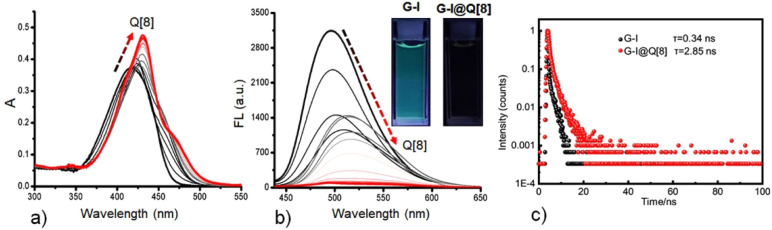
(a) UV-vis absorption and (b) fluorescence emission spectra obtained for G-I (10.0 µM of each) at increasing concentrations of the Q[8] host in an aqueous solution (pH 7.2) at 298 K (*λ*_ex_ = 400 nm). (c) Fluorescence decay traces of G-I and G-I@Q[8].

In aqueous solution, free G-I displayed a single absorption peak at ∼417 nm (Fig. 1a) and emitted blue fluorescence centered at ∼496 nm (Fig. 1b). Upon addition of the Q[8] host, the G-I solution exhibited a red-shifted absorption spectrum with enhanced intensity and significantly quenched fluorescence. Notably, the G-I@Q[8] complex showed a narrow absorption band at 431 nm, attributable to restricted rotational freedom of the guest within the Q[8] cavity. Concurrently, a broad absorption band emerged at longer wavelengths (450–500 nm), indicative of Q[8]-enhanced charge-transfer interactions. The fluorescence quenching of G-I upon Q[8] complexation confirmed the heavy-atom effect induced by the iodine substituents in the host–guest system. Supporting this mechanism, ^1^H NMR titration experiments revealed upfield shifts in the proton signals of the 4-iodophenyl groups (Fig. S1), confirming encapsulation of the terminal 4-iodophenyl moieties within the Q[8] cavity. Time-correlated single photon counting (TCSPC) indicated that a remarkably increased lifetime of G-I (from 0.34 ns to 2.85 ns) was achieved after encapsulation by the Q[8] hosts (Fig. 1c). To gain deeper insight into the photophysical origin of these changes, we calculated the radiative (*k*_r_) and non-radiative (*k*_nr_) decay rate constants (Table S1). The analysis shows that although both *k*_r_ and *k*_nr_ decrease upon encapsulation, the suppression of *k*_r_ is more pronounced, accounting for the observed emission quenching. Nevertheless, the overall excited-state decay rate (*k*_total_ = *k*_r_ + *k*_nr_) decreases due to the strong restriction of non-radiative pathways—a result of the rigidification of G-I within the Q[8] cavity—thereby resulting in the concurrent elongation of the fluorescence lifetime.

**Fig. 2:**
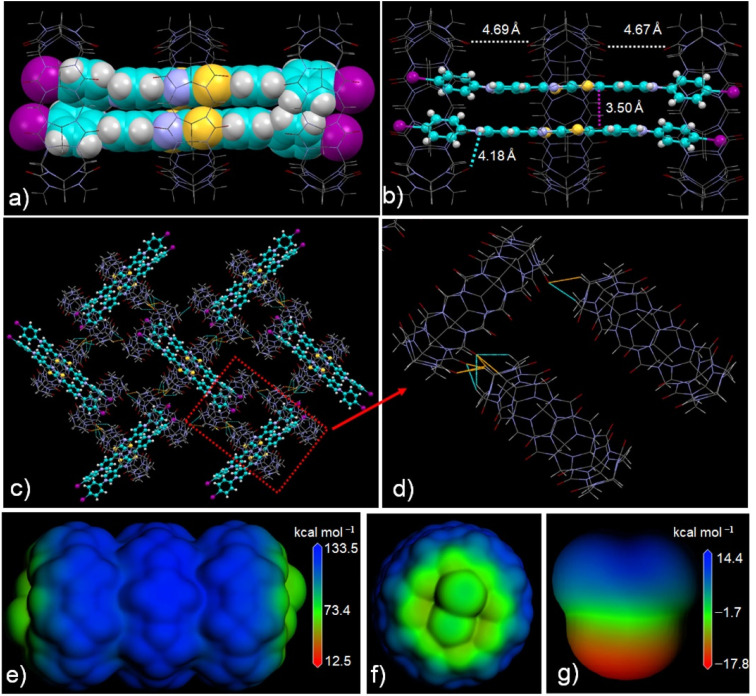
(a–d) X-ray crystal structure of G-I@Q[8]. (e–g) Electrostatic potential surface (ESP) calculations of G-I@Q[8] and HCHO (blue-color: electro positive region, green-color: near-zero ESP, red-color: electronegative region). ESP calculations were performed at the GGA/PBE level of theory with DMol3.

X-ray single-crystal diffraction analysis revealed that G-I guests form face-to-face dimers mediated by three Q[8] hosts through multiple hydrogen-bonding and ion–dipole interactions. As shown in Fig. 2a and b, the thiazolo[5,4-*d*]thiazole and phenyl moieties are encapsulated within the Q[8] cavity, while the pyridinium groups remain outside, consistent with the earlier ^1^H NMR observations. These results confirm that the host–guest binding mode of G-I@Q[8] remains identical in both solution and solid-state environments.

The distances between adjacent Q[8] hosts were measured to be 4.69 Å and 4.67 Å, respectively. Notably, the shortest distance between the portal carbonyl oxygen of the central Q[8] host and the positively charged nitrogen atoms of the pyridinium moiety was measured to be 4.18 Å, indicative of strong ion–dipole interactions. These findings demonstrate that the two terminal Q[8] hosts stabilize the guest molecules, acting as anchoring units that position the central Q[8] host near the thiazolo[5,4-*d*]thiazole group. Crucially, the face-to-face G-I dimer within the Q[8] cavity exhibits an interplanar separation of 3.50 Å—significantly closer than the 3.69 Å distance observed in our previously reported Q[8] complex with *p*-methylphenyl-substituted guests (Fig. S2). This reduced distance suggests enhanced π–π stacking interactions and stronger intermolecular electronic coupling between the dimerized G-I guests, which promotes intersystem crossing (ISC) in the guest molecules.^52^ As shown in Fig. S3, reactive oxygen species (ROS) generation assays revealed that the G-I@Q[8] complex exhibits significantly enhanced ^1^O_2_ production compared to the anisole-group-appended host–guest complex.^52^ Notably, G-I@Q[8] demonstrates a remarkably high quantum yield for ^1^O_2_ generation, superior to several recently reported pure organic photocatalysts. (Table S2). Consequently, the G-I@Q[8] system functions as a homogeneous supramolecular photocatalyst, enabling efficient sulfide oxidation (Fig. S4) and photodegradation of organic dyes (Fig. S5). Nevertheless, these represent universal characteristics among reported supramolecular photocatalysts. The discovery of novel properties in host–guest catalysts motivates further research expansion.

Fortunately, the successful acquisition of the crystal structure of the G-I@Q[8] complex provided critical insights into its supramolecular assembly behavior in the solid state, offering a foundation for exploring novel functionalities. As shown in Fig. 2c and d, strong hydrogen-bonding interactions occur between the portal carbonyl groups of Q[8] and the outer surfaces of adjacent Q[8] molecules within the solid-state assemblies. For example, hydrogen bonds form between methine/methylene (yellow/green color) groups on the Q[8] outer surface and the carbonyl oxygens of neighboring Q[8] molecules. These interactions drive the assembly of G-I@Q[8] into a multilayered two-dimensional framework featuring interconnected pore channels (Fig. S6). Electrostatic potential surface (ESP) calculations further revealed that the portal carbonyl regions of the Q[8] host are neutralized by the positively charged guest molecules, while the outer surface exhibits a slight increase in positive electrostatic potential (Fig. 2e and f). Based on our prior work with Q[*n*]-mediated outer-surface interactions, we explored the G-I@Q[8] system as a solid adsorbent for polar molecules such as formaldehyde (HCHO) (Fig. 2g).

**Fig. 3:**
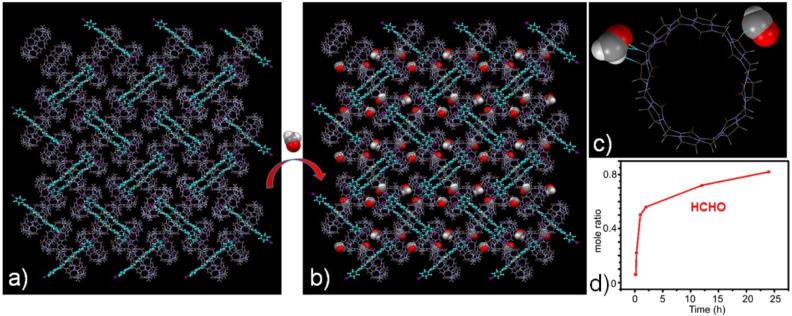
Packing structures of (a) G-I@Q[8] and (b) G-I@Q[8]-HCHO, viewed along the *a* axis. Packing structure showing (c) outer-surface interactions in the G-I@Q[8]-HCHO complex. (d) Time-dependent solid-vapor sorption plots of G-I@Q[8] for single-component vapors.

To characterize the adsorption behavior of the G-I@Q[8] system toward HCHO, single-component solid–vapor sorption experiments were conducted under ambient conditions (room temperature and atmospheric pressure; Fig. S7). The time-dependent molar ratio of HCHO to G-I@Q[8] was quantified *via*^1^H NMR analysis. As shown in Fig. 3d, the HCHO adsorption capacity of the G-I@Q[8] solid powder progressively increased over time and reached saturation after 24 hours. At the saturation point, the molar ratio of HCHO to G-I@Q[8] was approximately 0.84, demonstrating the system's high adsorption efficiency for HCHO. Notably, single crystals suitable for structural analysis were successfully obtained by immersing G-I@Q[8] complex crystals in a formaldehyde solution for several days. X-ray single-crystal diffraction analysis revealed that the G-I@Q[8]-HCHO complex crystallized in the space group *P*2_1_/*n*—identical to the space group of the original G-I@Q[8] complex (Tables S3 and S4). This observation confirms that the assembly of the G-I@Q[8] complex remains unaltered upon formaldehyde adsorption. As illustrated in Fig. 3b and c, the crystal structure of G-I@Q[8]-HCHO unambiguously shows that HCHO molecules are localized around the periphery of the G-I@Q[8] units *via* outer-surface interactions, including C–H⋯O hydrogen bonds and ion–dipole interactions. Significantly, when viewed along the crystallographic *a*- and *b*-axis directions of the G-I@Q[8]-HCHO packing structure, a substantial number of entrapped HCHO molecules are observed within the supramolecular framework (Fig. S8).

**Fig. 4:**
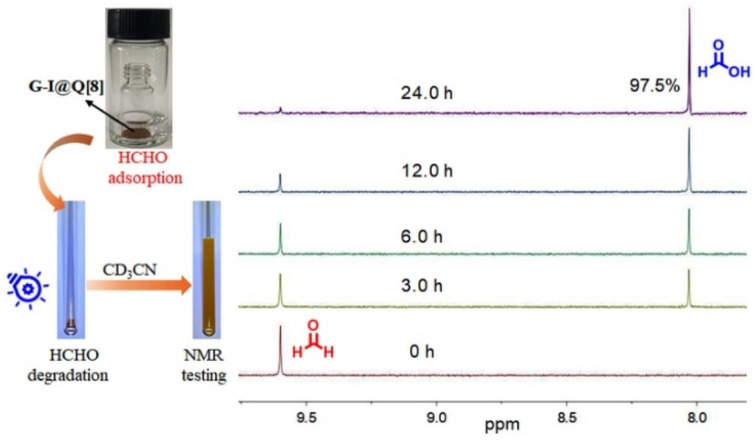
Schematic illustration of HCHO adsorption, degradation, and NMR testing by G-I@Q[8] (left). Time-dependent ^1^H NMR spectrum of G-I@Q[8]-HCHO under blue LED light irradiation at room temperature (right).

Subsequently, the photocatalytic performance of the G-I@Q[8] complex for HCHO was investigated. The photo-degradation process of adsorbed HCHO by the G-I@Q[8] solid was monitored *via*^1^H NMR spectroscopy, using CD_3_CN as the extraction solvent for analyte recovery. As shown in Fig. 4, the G-I@Q[8]-HCHO composite demonstrated exceptional photocatalytic activity, achieving 97.5% conversion of adsorbed HCHO to formic acid (HCOOH) within 24 hours under blue light irradiation(440–450 nm; 20 W). Notably, the G-I@Q[8] system retained its catalytic functionality even under natural sunlight (Fig. S9), which can be attributed to the strong light absorption of the G-I@Q[8] complex in the 400–600 nm range (Fig. 1a). Although both pristine Q[8] and G-I exhibited intrinsic HCHO adsorption capabilities under identical conditions, their individual photocatalytic degradation efficiencies were negligible (Fig. S10–S13). These findings collectively establish G-I@Q[8] as a bifunctional supramolecular photocatalyst that synergistically integrates adsorption and catalytic degradation capabilities for efficient HCHO removal. Most interestingly, the G-I@Q[8] complex, when used as a heterogeneous photocatalyst in CH_3_CN, displayed fast degradation of HCHO to HCOOH compared to the solid-state system (Fig. S14). Furthermore, we have systematically demonstrated that high catalytic efficiency requires specific wavelength matching the absorption of G-I@Q[8] and a properly sized host cavity, as evidenced by control experiments under varied light conditions and with different macrocyclic hosts (Fig. S15–S20).

**Fig. 5:**
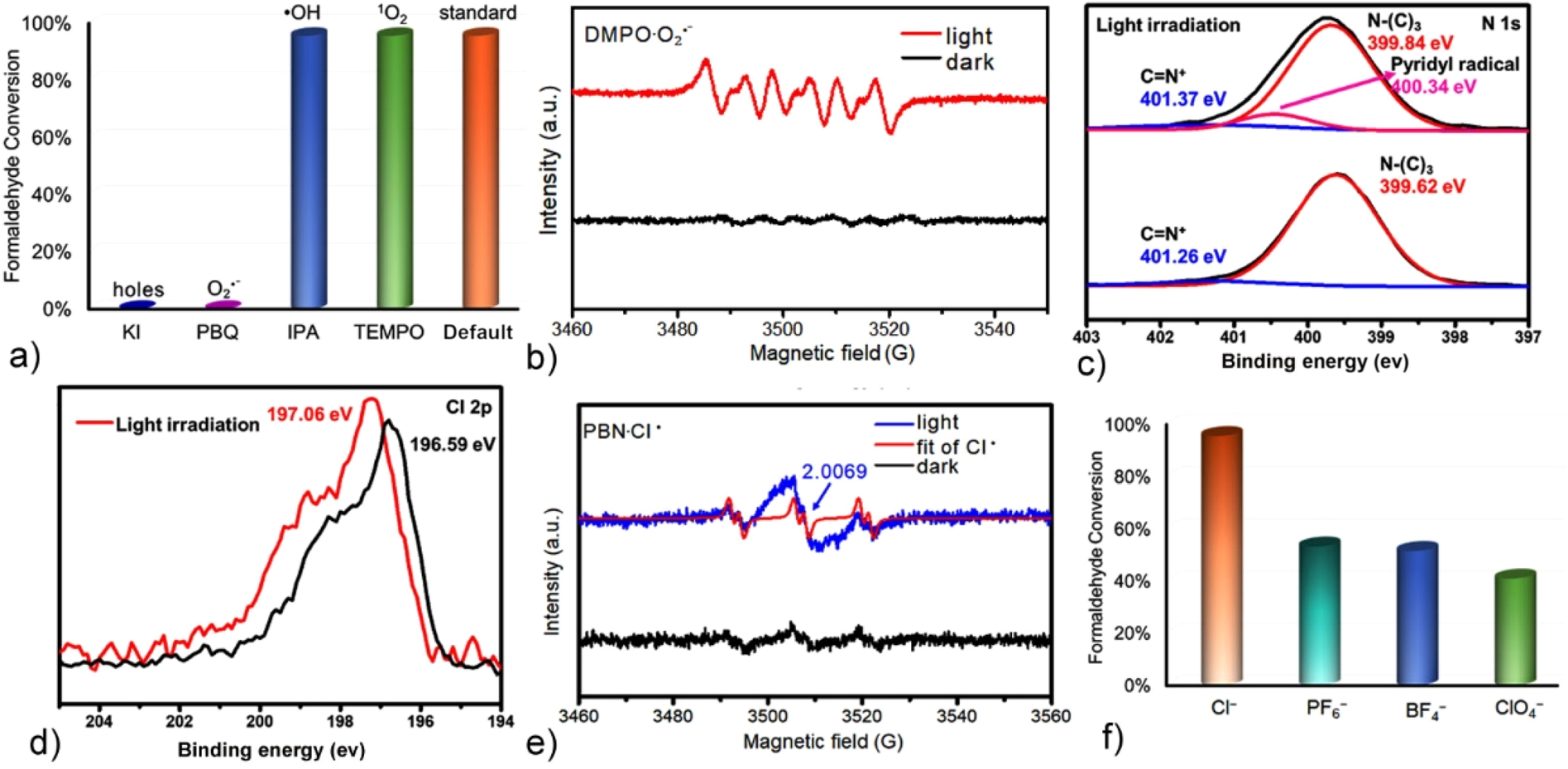
(a) Quenching experiments of HCHO photooxidation in CH_3_CN using G-I@Q[8] as a heterogeneous catalyst. (b) DMPO spin-trapping EPR spectra for DMPO O_2_˙^−^ over G-I@Q[8] under light irradiation. XPS spectra corresponding to (c) N 1s and (d) Cl 2p in the G-I@Q[8] systems before and after irradiation with a light source. (e) PBN spin-trapping EPR spectra for PBN Cl˙ over G-I@Q[8] under light irradiation. (f) Control experiments of HCHO photooxidation in CH_3_CN with G-I@Q[8] containing different counteranions as heterogeneous catalysts.

Our previous studies demonstrated that Q[8]-encapsulated TTDP derivative dimers exhibit exceptional singlet oxygen (^1^O_2_) generation efficiency in aqueous media.^52^ To elucidate the aerobic oxidation mechanism and identify the reactive oxygen species (ROS) involved in the present photocatalytic process, a series of quenching-controlled experiments were performed in CH_3_CN, where the G-I@Q[8] complex acted as a heterogeneous photocatalyst.^55,56^ The addition of propan-2-ol (IPA, a hydroxyl radical scavenger) and 2,2,6,6-tetramethylpiperidin-1-oxyl (TEMPO, a singlet oxygen quencher) showed no inhibitory effect on the reaction kinetics, thereby excluding the involvement of hydroxyl radicals (˙OH) and singlet oxygen (^1^O_2_). Conversely, complete reaction suppression was achieved upon introducing 1,4-benzoquinone (PBQ, a superoxide anion-specific scavenger) and KI (a hole quencher), which unequivocally confirmed the dominant roles of superoxide anion radicals (O_2_˙^−^) and photogenerated holes in the oxidation pathway (Fig. 5a and S21–S24). This mechanism was further supported by light-triggered electron paramagnetic resonance (EPR) spectroscopy: irradiation of the G-I@Q[8] system produced a distinctive six-line EPR signal with a 1 : 1 : 1 : 1 : 1 : 1 intensity ratio, providing direct evidence for O_2_˙^−^ generation (Fig. 5b).^57–59^ These results provided unequivocal proof that the heavy atom effect of the iodophenyl groups in the guest plays a key role in promoting the G-I@Q[8] complex to generate O_2_˙^−^ rather than ^1^O_2_.

X-ray photoelectron spectroscopy (XPS) analysis of the G-I@Q[8] catalyst was conducted to elucidate its elemental composition and valence state. Fig. S25 shows XPS spectra revealing the presence of C, N, O, S, I, and Cl in the catalyst. After light irradiation, a new peak at 400.34 eV emerged in the N 1s core-level spectrum (Fig. 5c), indicating the nitrogen atom of the pyridyl radical on the TTDP moieties. Notably, a higher energy shift of Cl^−^ from 196.59 eV to 197.06 eV was observed in the spectra (Fig. 5d),^60^ while no significant binding energy changes were observed for the C 1s, O 1s and I 3d orbitals following irradiation (Fig. S26). These spectroscopic changes suggest that the pyridinium moieties in G-I@Q[8] possess strong electron-accepting capabilities, with the Cl^−^ counteranions functioning as electron donors. Importantly, the generation of Cl˙ was further confirmed by the EPR spectra in the presence of *N-tert*-butyl-α-phenylnitrone (PBN) as a trapping agent (Fig. 5e).^61^ As a comparative study, other anions such as PF_6_^−^, BF_4_^−^, and ClO_4_^−^ were used as counteranions instead of Cl^−^ in the G-I@Q[8] system, and a lower efficiency for HCHO-to-CHCOOH conversion was observed (Fig. 5f and S27–29). These results suggested that Cl^−^ counteranions play a crucial role in the photocatalytic process of the G-I@Q[8] complex as a heterogeneous catalyst. Additionally, G-I@Q[8] functions as a heterogeneous catalyst with excellent stability and versatility (Fig. S30). It also demonstrates efficient degradation of CEES and oxidation of benzyl alcohol (Fig. S31 and 32).

To further understand the pathway of O_2_˙^−^ generation of the G-I@Q[8] system, the photocatalytic oxidation of benzylamine (a typical photocatalytic reaction mediated by O_2_˙^−^) using the G-I@Q[8] complex as a heterogeneous catalyst in CH_3_CN solution was performed.^62^ This experiment demonstrated that the Cl^−^ counteranions donate electrons to reduce O_2_ to O_2_˙^−^ (Fig. S33). However, the oxidation of benzylamine could not proceed when the G-I@Q[8] complex was used as a homogeneous catalyst in aqueous media under identical reaction conditions (Fig. S34). From a structural viewpoint, although the G-I@Q[8] complex retains the same assembly fashion in both solution and solid-state phases, the key difference lies in the hydration of Cl^−^ counteranions in aqueous solution.^63^ This hydration process inhibits the electron-donating ability of Cl^−^ to the pyridinium atoms in G-I (Scheme 1).

These results collectively indicate that Cl^−^ counteranions play a key role in facilitating electron transfer for O_2_˙^−^ generation when the G-I@Q[8] complex functions as a heterogeneous catalyst.

Interestingly, spectral changes of 9,10-anthracenediyl-bis(methylene)dimalonic acid (ABDA, a ^1^O_2_ indicator) revealed that ^1^O_2_ was generated when the G-I@Q[8] complex acted as a homogeneous catalyst in aqueous solution (Fig. S3), while no spectral change was observed (indicating no ^1^O_2_ generation) when the G-I@Q[8] complex functioned as a heterogeneous catalyst in CH_3_CN solution (Fig. S35). Consequently, we have demonstrated a unique phase-dependent photocatalyst capable of selectively controlling ^1^O_2_ and O_2_˙^−^ generation in this work (Scheme 1). This finding provides critical mechanistic insights into the selective regulation of ROS generation in photocatalytic systems.

**Fig. 6:**
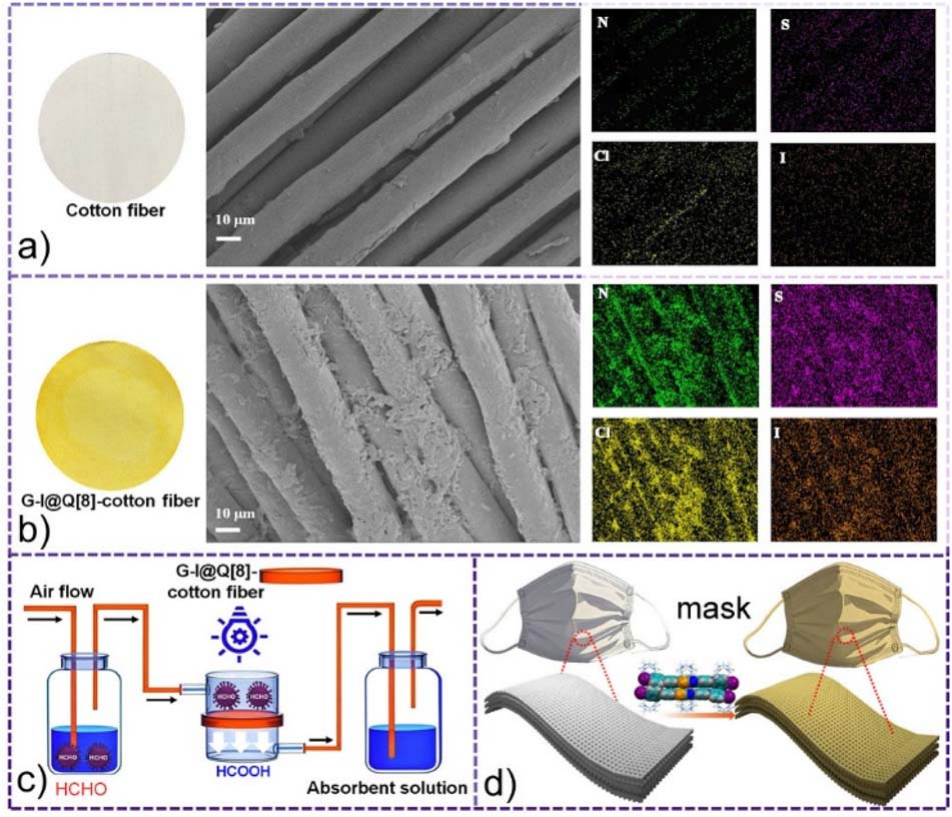
(a and b) Comparison of the SEM images between parent untreated fibers and the G-I@Q[8]-cotton fiber composite; scale bar: 10 µm. Elemental mapping of untreated fibers and the G-I@Q[8]-cotton fiber composite; scale bar: 10 µm; nitrogen: green, sulfur: purple, chlorine: yellow, and iodine: orange. (c and d) Schematic representation of G-I@Q[8]-cotton for HCHO removal.

Based on the above observations, we developed a scalable strategy to immobilize the G-I@Q[8] complex onto cotton fibers—a high-surface-area substrate that synergistically enhances photocatalytic performance by effectively dispersing the photoactive phase, improving the separation of photogenerated carriers, and facilitating the adsorption of target pollutants near the photocatalytic reaction sites. The composite was synthesized *via* a one-step impregnation method (Fig. 6a and b): cotton fibers were immersed in an aqueous G-I@Q[8] solution (1.0 mM) for 60 minutes, followed by thermal annealing at 60 °C for 60 minutes to remove residual solvent. A distinct chromatic transition from white to yellow confirmed the successful supramolecular immobilization of G-I@Q[8] on the fiber surface. The resulting composite was designated as G-I@Q[8]-cotton.

To further characterize the loading of G-I@Q[8], scanning electron microscopy (SEM) analysis was performed. As shown in Fig. 6a, the interwoven structure of the pristine cotton fibers was clearly observed, with smooth fiber surfaces. After immobilization of the G-I@Q[8] complex (Fig. 6b), the fiber surfaces became uniformly covered with G-I@Q[8] particles. Furthermore, elemental mapping of the G-I@Q[8]-cotton composite revealed homogeneous distribution of N, S, Cl, and I across the fiber surface, with no significant aggregation. These results collectively demonstrate the successful preparation of the G-I@Q[8]-cotton fabric *via* the one-step impregnation method.

As shown in Fig. 6c, S36 and S37, a custom-designed reactor system was constructed to evaluate the adsorption–degradation performance of G-I@Q[8]-cotton fibers toward HCHO.

Experimental results revealed that both untreated parent cotton fibers and G-I-cotton fibers exhibited negligible adsorption–degradation activity, leading to immediate HCHO breakthrough. In contrast, the G-I@Q[8]-cotton fibers achieved sustained and efficient HCHO removal. These findings demonstrate that supramolecular functionalization of fibrous materials through the loading strategy significantly enhances their dual adsorption–catalytic degradation capabilities for HCHO. Consequently, G-I@Q[8]-cotton fibers show promising potential for application in masks as integrated adsorption–photocatalytic materials, as conceptually illustrated in Fig. 6d. Importantly, the photocatalytic degradation of HCHO by G-I@Q[8]-cotton under both blue light and natural sunlight enables convenient mask regeneration, thereby promoting reusable functionality.

## Conclusions

We have developed a novel photosensitizer through the dimerization of the guest molecules within Q[8] cavities, which demonstrates phase-dependent selective control over ^1^O_2_ and O_2_˙^−^ generation. Capitalizing on the high affinity of the Q[8] host's outer surface for HCHO in the solid state, this host–guest assembly serves as a dual-functional adsorbent–photocatalytic system, establishing an efficient platform for simultaneous HCHO capture and degradation. This study not only presents a novel strategy for constructing multifunctional organic photocatalysts with selective ROS generation control, but also advances the application of Q[*n*]-based supramolecular assemblies by synergistically leveraging both cavity confinement effects and outer-surface interactions.

## Author contributions

X.-L. N. designed and conducted the project. H. N., J. T., and Y. L. performed the synthesis and characterization. H. N. and X.-L. N. prepared the manuscript.

## Conflicts of interest

The authors declare no competing interests.

## Supplementary Material

SC-017-D5SC06904A-s001

SC-017-D5SC06904A-s002

## Data Availability

CCDC 2473180 and 2473294 contain the supplementary crystallographic data for this paper.^64*a*,*b*^ The data supporting the article have been included as a part of the supplementary information (SI). Supplementary information is available. See DOI: https://doi.org/10.1039/d5sc06904a.
