# Supplementary material for: A cucurbit[8]uril-triggered ionic photosensitizer in solution and solid states: selective control of 1O2 and O2˙− generation
Source: Chem Sci. 2026 Jan 6;17(8):4137–44. doi: 10.1039/d5sc06904a (PMC12771535; doi:10.1039/d5sc06904a)
Supplement: SC-017-D5SC06904A-s001 [file SC-017-D5SC06904A-s001.pdf]

Supplementary Information (SI) for Chemical Science.
This journal is © The Royal Society of Chemistry 2025

Supporting Information

Cucurbit[8]uril-Triggered Ionic Photosensitizer in Solution and Solid-state: Selective Control of $^1O_2$ and $O_2^{\bullet -}$ generations

Haigen Nie, Jiao Tan, Yi Luo, and Xin-Long Ni*

Key Laboratory of Chemical Biology and Traditional Chinese Medicine Research (Ministry of Education of China), Key Laboratory of the Assembly and Application of Organic Functional molecules of Hunan Province
College of Chemistry and Chemical Engineering, Hunan Normal University, Changsha 410081 (China)
*Correspondence to longni333@163.com

General instrumentation and methods.

^{1}H NMR (500 MHz), ^{1}H NMR (700 MHz) and ^{13}C NMR (176 MHz) spectra were obtained on Bruker ADVANCE-500 and Bruker ADVANCE-700 spectrometers, respectively. UV/vis absorption spectra were recorded on a Shimadzu UV-3600 spectrometer. Fluorescence emission spectra were recorded on a Hitachi F-7000 spectrometer. X-ray data were taken on an Agilent Supernova X-ray diffractometer equipped with a large area CCD detector. X-ray photoelectron spectra (XPS) were detected using Thermo Scientific K-Alpha instrument with Al Kα ($h\nu$ = 1486.6 eV) radiation. Electron paramagnetic resonance (EPR) spectra were recorded on a JES-FA200 spectrometer. The transient absorption spectra was obtained by Femto-TA100 transient absorption spectrometer. The power of the LED light source is 20W. Unless otherwise noted, materials obtained from commercial suppliers were used without further purification.

Preparation of heterogeneous photocatalysts.

The **G-I@Q[8]** was obtained from encapsulation of **G-I** within Q[8] host in water at room temperature. Typically, after dissolving chloride salt of **G-I** (1 eq.) in deionized water, Q[8] (1.5 eq.) was added, and the mixture was stirred in a round-bottomed flask at 100 °C until the solution became transparent to obtain the **G-I@Q[8]** solution. The solid-state **G-I@Q[8]** was obtained by air-drying the **G-I@Q[8]** solution. For the convenience of description, the amount of host–guest complex is equivalent to the amount of guest molecules contained.

Detection of $^{1}O_2$ production in aqueous/CH_3CN solution.

Compound 9,10-anthracenediylbis-(methylene)dimalonic acid (ABDA) was used as an indicator for detection of $^{1}O_2$ in the aqueous/CH_3CN solution. The aqueous/CH_3CN solutions of 1.5×10^{-4} M ABDA with 1.5×10^{-6} M **G-I@Q[8]**、**G1@Q[8]** or **G-I** irradiated by white LED light source, respectively. The absorption change of the mixed solution at 378 nm were measured by the UV-vis absorption spectrophotometer.

Electron paramagnetic resonance (EPR) test.

The existence of $O_2^{\bullet-}$ was detected by EPR measurements. Typically, 40 μL DMPO in 1 mL acetonitrile was mixed with 0.5 mL **G-I@Q[8]** suspension (1 mg/1 mL). From the obtained mixture, 300 μL was taken and added into the EPR tube. EPR signals were recorded for the sealed capillary both in the absence and presence of light. For irradiation, a 300 W xenon lamp with 440 nm cutoff was used.

The existence of Cl· was detected by EPR measurements. Using N-benzylidene-tert-

butylamine-N-oxide (PBN) as the free radical scavenger, the catalyst was dispersed in CH_3CN to detect the production of chlorine radical (Cl·). Specifically, **G-I@Q[8]** was dispersed in a CH_3CN solution (1 mg/1 mL). After that, 50 μL of the above suspension was mixed with PBN. The mixture was characterized by an EPR spectrometer at room temperature, and the spectra were collected after illumination for a certain time.[1]

General Procedure for the Oxidation of Benzylamine.

The catalyst (**G-I@Q[8]**, 1×10^{-4} mmol) was added in D_2O (0.5 mL) containing benzylamine (2.0×10^{-2} mmol) in a NMR tube. After purging with O_2 for 15 min, the mixture was irradiated by blue LED light source for 4 hours. The conversion and selectivity were determined by the integral of the ^1H NMR signals.

Formaldehyde Adsorption Test.

A 2.0 mL open vial containing 30.0 mg of **G-I@Q[8]** was placed in a sealed 20.00 mL vial containing 1.5 mL of Formaldehyde. Prior to the measurements, the **G-I@Q[8]** were heated at 100 °C for 24 hours to remove any surface-physically adsorbed vapor. Subsequently, 3.0 mg of **G-I@Q[8]** was weighed each time and dispersed ultrasonically in CD_3CN containing pyrazine as an internal standard. The amount of formaldehyde adsorbed by **G-I@Q[8]** was determined by measuring the changes in the proton nuclear magnetic resonance (^1H NMR) spectrum before and after the adsorption process.

Formaldehyde Adsorption Test comparison.

G-I and Q[8] formaldehyde adsorption experiment: under the same test conditions.

Formaldehyde Degradation Test.

Separately weigh 3.0 mg of **G-I@Q[8]** solid, which has adsorbed formaldehyde, into NMR tubes, and illuminate the solids under sunlight/blue light for 24 hours, respectively. Then, add 0.5 mL of CD_3CN and subject the mixture to ultrasonic treatment for 20 minutes. The degradation products of formaldehyde were determined through ^1H NMR.

Formaldehyde Degradation Test comparison.

G-I and Q[8] formaldehyde degradation experiment: under the same test conditions.

Phenol reagent synthesis.

Formaldehyde (HCHO) content testing was conducted using the national standard method. Firstly, the preparation of the phenol reagent (3-Methyl-2-benzothiazolinonehydrazone Hydrochloride Hydrate: MBTH) was carried out, the absorbent stock solution was prepared by dissolving 0.1 g of phenol reagent in a 100

mL volumetric flask. Deionized water was added to the flask to dissolve and fix the volume, and the solution was stored in a refrigerator to stabilize for 3 days. 5 mL of absorbent stock solution was mixed with 95 mL of deionized water to obtain an absorbent solution. Then, 1% ferric ammonium sulfate solution was prepared. In detail, 1.0 g of ferric ammonium sulfate was dissolved in 100 mL of 0.1 mol/L HCl. Then 0.4 mL of 1% ferric ammonium sulfate solution was added to absorbent solution and shaken well. The absorbance was measured at 630 nm using deionized water as a reference. The principle is that HCHO in air reacts with a phenol reagent to form zine oxidized by iron ions in an acidic solution to form a blue-green compound. The color of the compound is proportional to the formaldehyde content. That is, the darker the color, the more formaldehyde content; the lighter the color, the lower the formaldehyde content. The quantitative test is carried out by UV-vis (Figure. S35).[2]

Designed experimental setup for formaldehyde absorption-degradation measurement.

The experimental setup for formaldehyde absorption-degradation measurement was built in our laboratory. First, a home-made permeation test reactor was assembled by clamping untreated fiber, **G-I-cotton** or **G-I@Q[8]-cotton** fiber composite (3 cm × 3 cm) between two quartz devices. The setup was illuminated with a LED blue light (440-450 nm) during use. Next, an air flow was passed through the cell containing formaldehyde. This air flow, containing formaldehyde, then swept through the absorption-degradation test reactor. A cell containing phenol reagent absorbent solution was used to collect the undegraded formaldehyde. Take 3.0 mL absorption solution every 30 minutes, add 240 μL 1% (w/v) ammonium ferric sulfate solution as chromogenic agent, shake well, and stand in the dark for 15 minutes to complete the color reaction. The absorbance was measured at 630 nm ~ 645 nm using a UV-vis spectrophotometer.

Synthesis and characterization

Synthesis of G-I guest

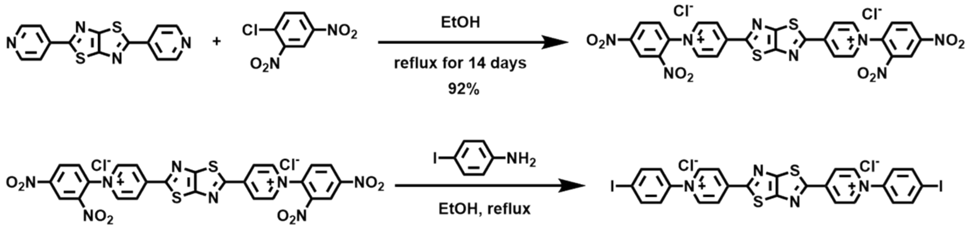

Dipyridinium thiazolo[5,4-d]thiazole (700 mg, 2.36 mmol) and 2,4-dinitrochlorobenzene (7.18 g, 35.44 mmol) was added to 300 mL EtOH. The mixture was stirred and under reflux for 14 days. The mixture was then cool to room temperature and the solvent was evaporated under reduced pressure to 10 mL, and then 40 mL acetone was added. The solid was obtained by centrifugation. The crude product was washed by acetone (30 mL×3). The residual solvent was evaporated in vacuum to afford the 1,1'-bis(2,4-dinitrophenyl)-dipyridinium thiazolo[5,4-d]thiazole dichloride (1.52 g, 92 %) as a brown solid. Then, a solution of 1,1'-bis(2,4-dinitrophenyl)-dipyridinium thiazolo[5,4-d]thiazole dichloride (130 mg, 0.185 mmol) and three equivalents p-Iodoaniline were refluxed in 100 mL ethanol for 24 hours. The mixture was then cool to room temperature and the solvent was evaporated under reduced pressure to 5 mL. The concentrated reaction mixture was mixed with 30 mL acetone, centrifugated, decanted and a solid was collected. The crude product was washed by acetone (25 mL×3). The residual solvent was evaporated in vacuum to afford the **G-I** (114.5 mg, 80 %) as a brown solid.

G-I: ^{1}H NMR (500 MHz, D_2O) δ 9.23(d, J = 10.0 Hz, 4H), 8.79 (d, J = 10.0 Hz, 4H), 8.13 (d, J = 10.0 Hz, 4H), 7.56 (d, J = 10.0 Hz, 4H). ^{13}C NMR (176 MHz, DMSO-d_6) δ 167.19 (s), 157.96 (s), 148.69 (s), 147.91 (s), 144.00 (s), 141.01 (s), 128.74 (s), 126.09 (s), 100.96 (s).

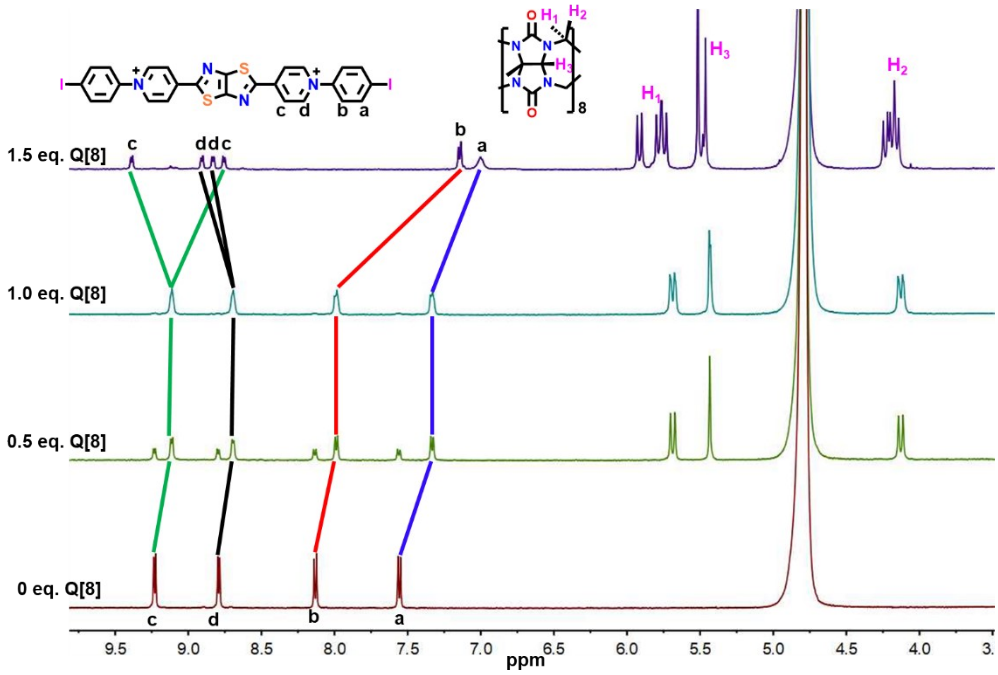

Figure S1. ^{1}H NMR spectra obtained for **G-I** (1.0 mM, D_2O) in the presence of different concentrations of the Q[8] host.

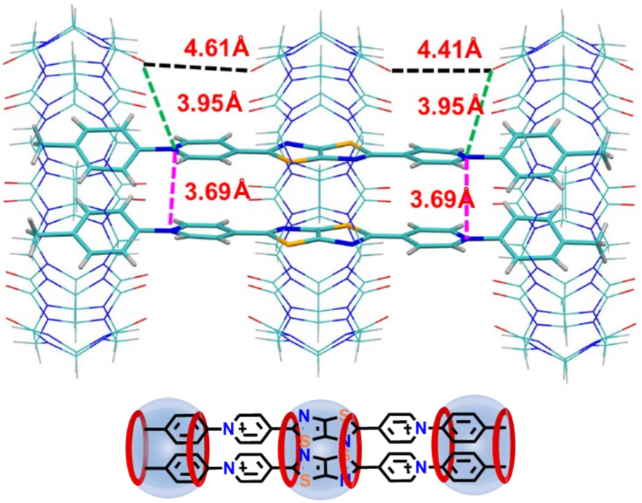

Figure S2. X-ray crystal structure of Q[8] complex with *p*-methylphenyl-substituted guests.[3]

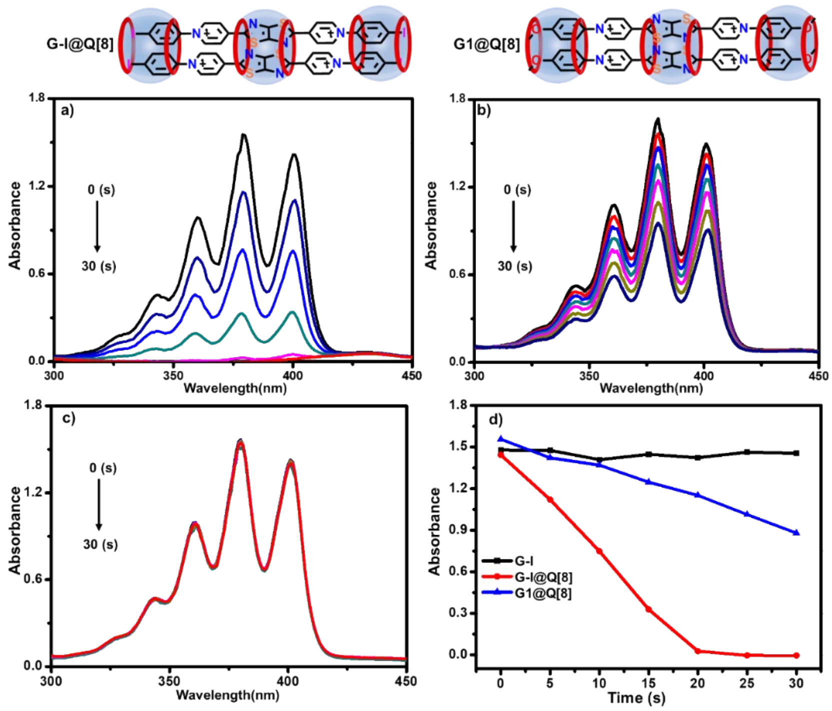

Figure S3. UV-vis spectral changes of 9,10-anthracenediyl-bis(methylene)dimalonic acid (ABDA) (1.5×10^{-4} M) in the presence of a) **G-I@Q[8]**, b) **G1@Q[8]**,[3] and c) **G-I** (each of 1.5×10^{-6} M for guest) under white light irradiation in water at room temperature. d) Irradiation-time dependent absorbance changes of ABDA at 378 nm.

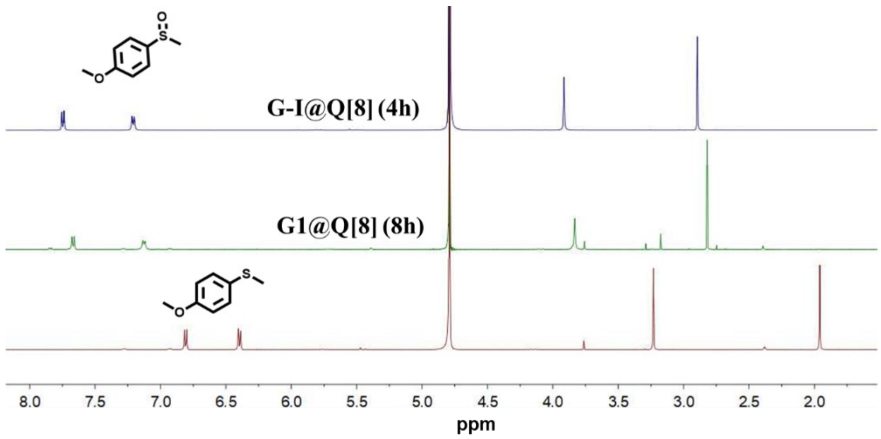

Figure S4. ^{1}H NMR spectra of 4-Methoxythioanisole (4.0×10^{-2} M) in the presence of **G-I@Q[8]**, and **G1@Q[8]** (each of 2.0×10^{-4} M for guest) as photocatalyst under white LED light irradiation in D_2O for 4 h and 8 h at room temperature, respectively.

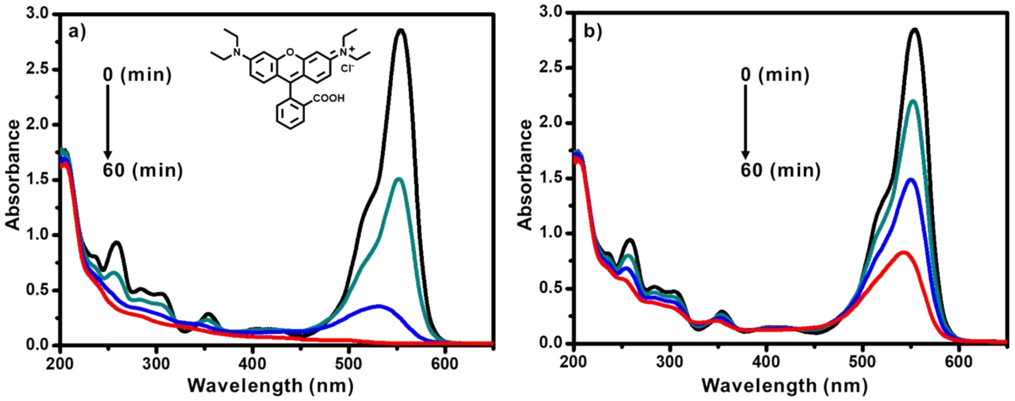

Figure S5. UV-vis spectra of RB (1.5×10^{-4} M) in the presence of a) **G-I@Q[8]** and b) **G1@Q[8]** (each of 1.5×10^{-6} M for guest) under white LED light irradiation.

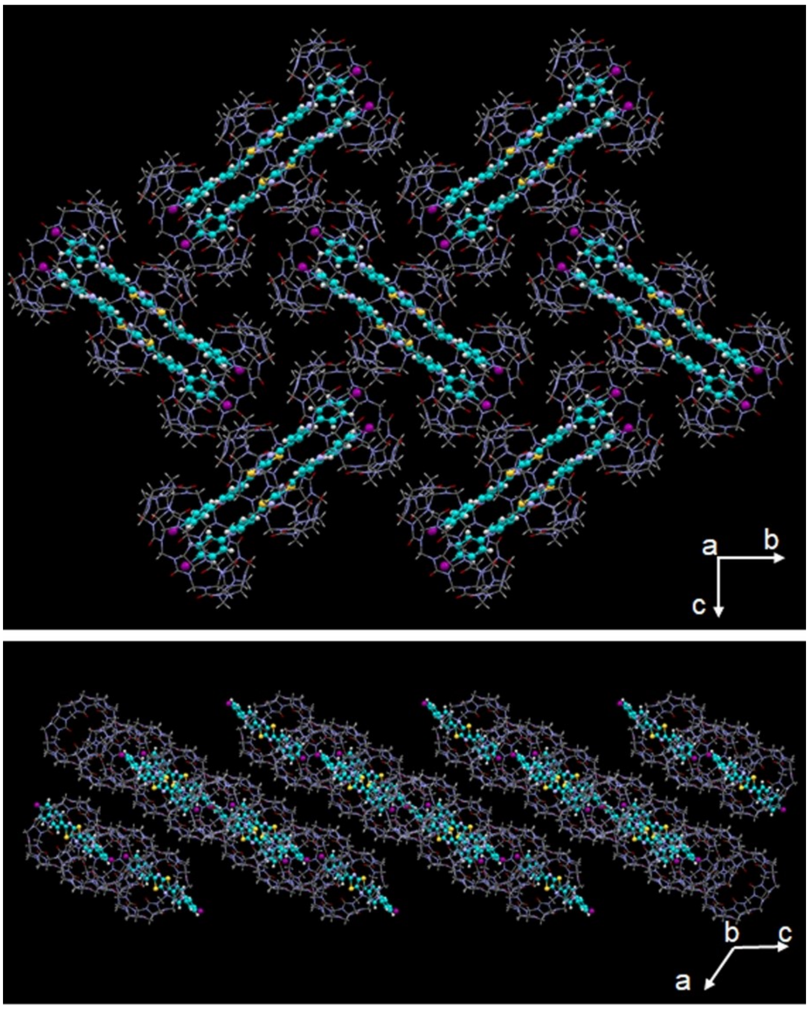

Figure S6. Outer-surface interactions drive the assembly of **G-I@Q[8]** into a multilayered two-dimensional frameworks

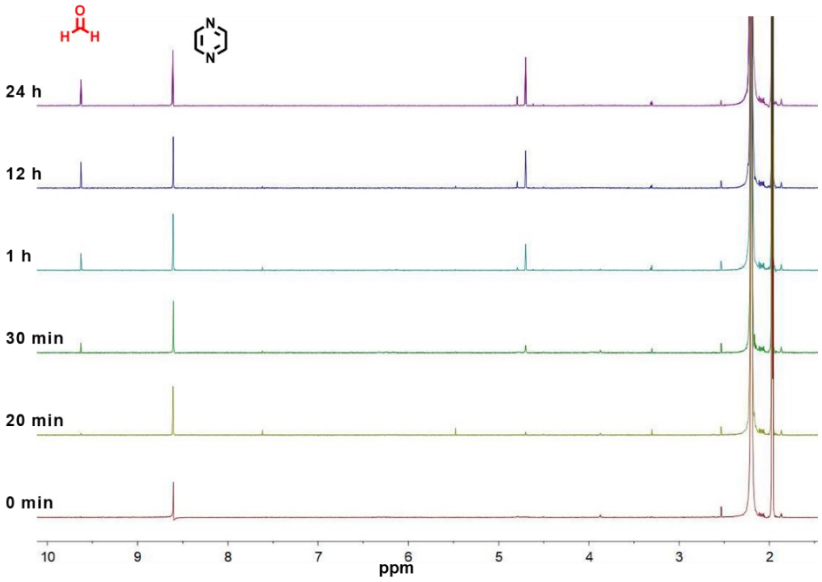

Figure S7. Time-dependent ^{1}H NMR spectrum of **G-I@Q[8]** after adsorption of **CH_2O** vapor (pyrazine was used as the internal reference compound).

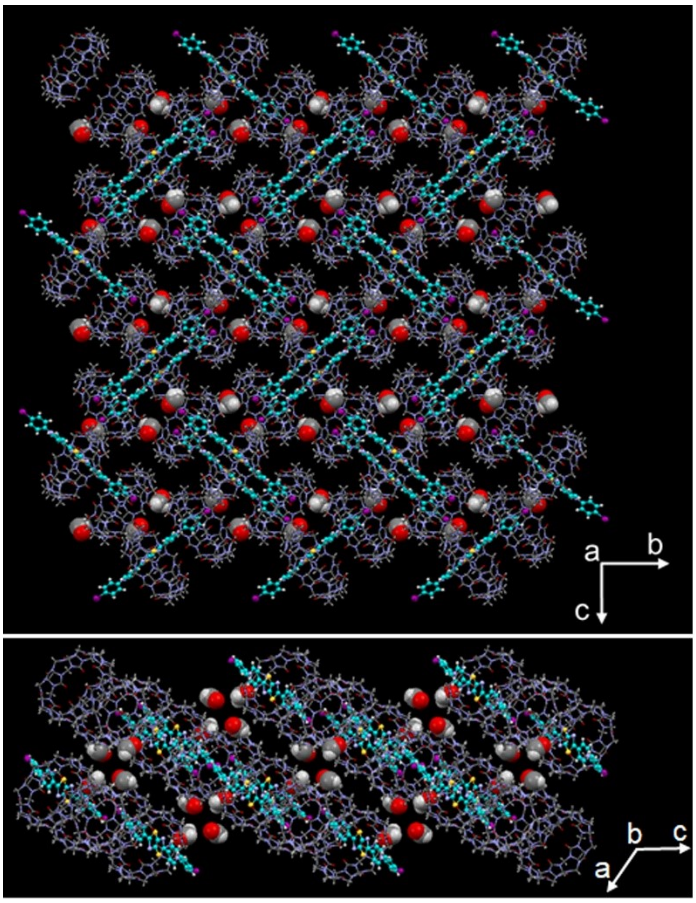

Figure S8. Outer-surface interactions drive the assembly of **G-I@Q[8]** into a multilayered two-dimensional frameworks for HCHO capture.

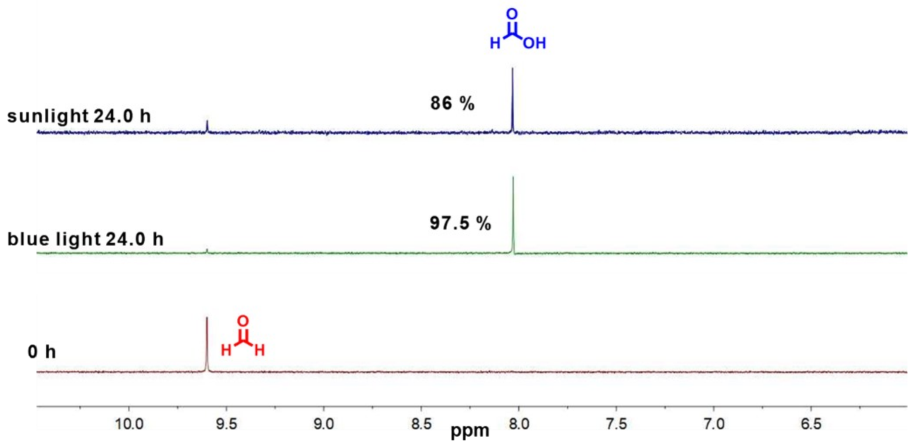

Figure S9. ^{1}H NMR spectrum of **G-I@Q[8]-HCHO** under blue LED light and sunlight irradiation for 24 h at room temperature.

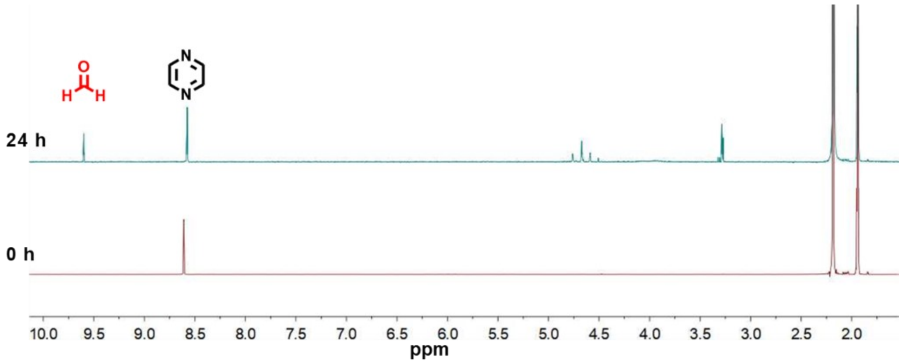

Figure S10. ^{1}H NMR spectrum of **Q[8]** after adsorption of **CH_2O** vapor for 24 h (pyrazine was used as the internal reference compound).

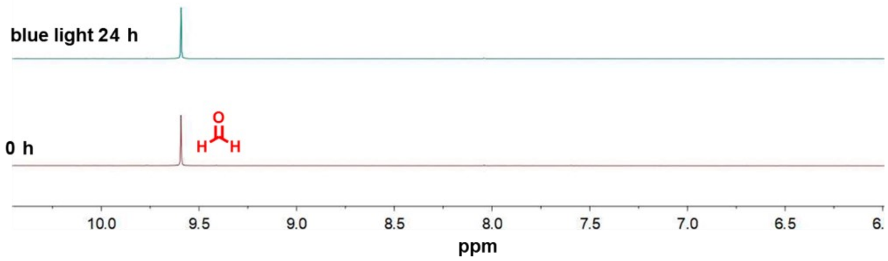

Figure S11. ^{1}H NMR spectrum of **Q[8]-CH_2O** under blue LED light irradiation for 24 h at room temperature.

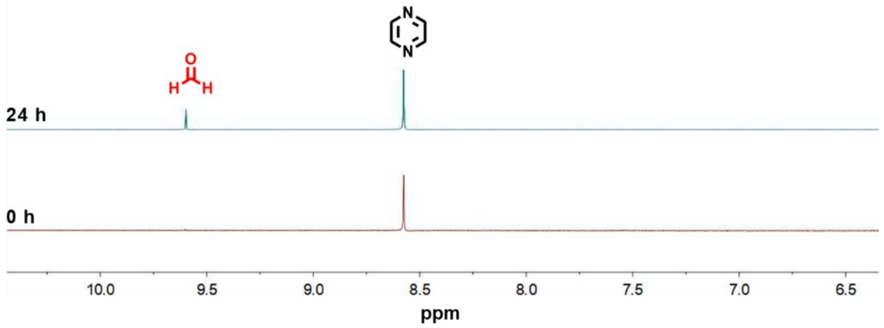

Figure S12. ^{1}H NMR spectrum of **G-I** after adsorption of **CH_2O** vapor for 24 h (pyrazine was used as the internal reference compound).

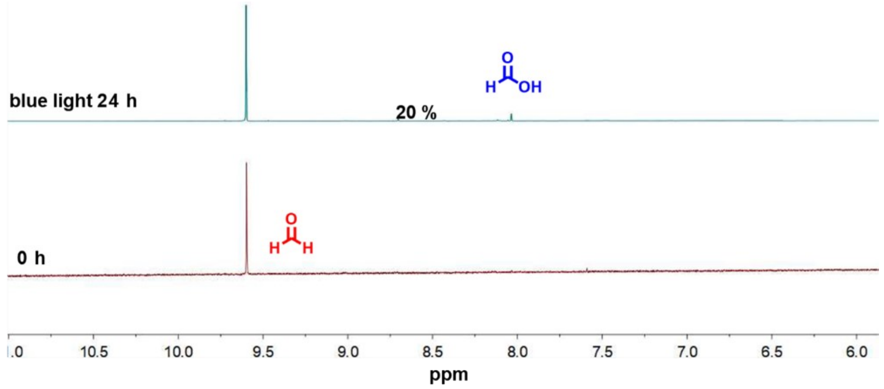

Figure S13. ^{1}H NMR spectrum of **G-I-CH_2O** under blue LED light irradiation for 24 h at room temperature.

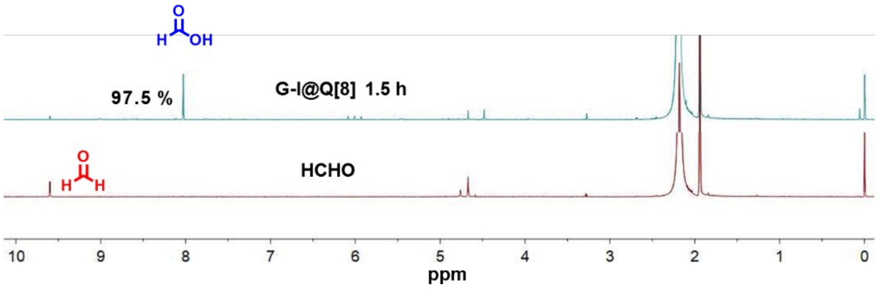

Figure S14. ^{1}H NMR spectrum of **HCHO** (2.0×10^{-2} mmol) in the presence of **G-I@Q[8]** (1.0×10^{-4} mmol) under blue LED light irradiation in CD_3CN for 1.5 h at room temperature.

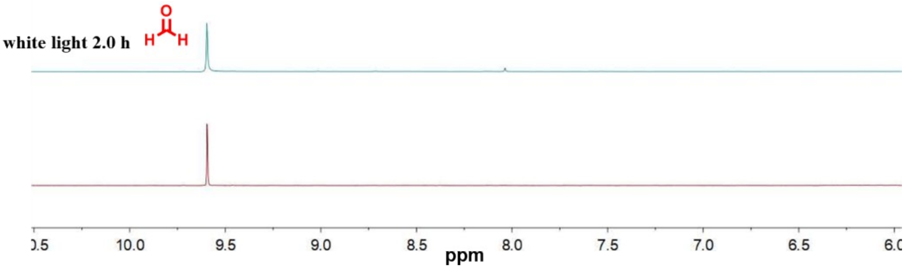

Figure S15. ^{1}H NMR spectrum of **HCHO** (2.0 × 10^{-2} mmol) in the presence of **G-I@Q[8]** (1.0 × 10^{-4} mmol) under white LED light irradiation in CD_3CN for 1.5 h at room temperature.

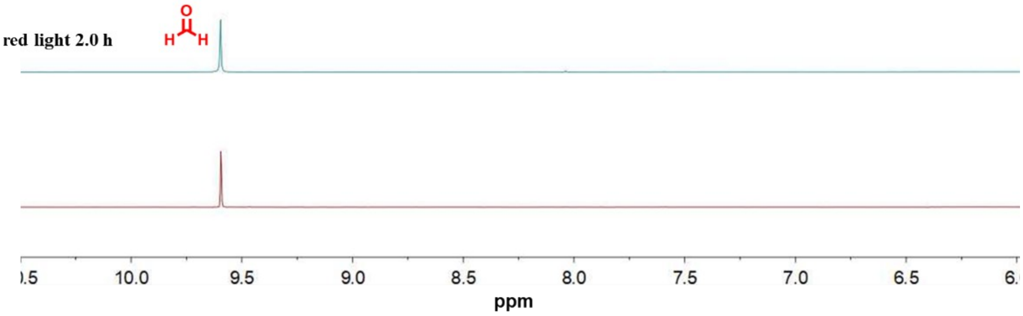

Figure S16. ^{1}H NMR spectrum of **HCHO** (2.0 × 10^{-2} mmol) in the presence of **G-I@Q[8]** (1.0 × 10^{-4} mmol) under red LED light irradiation in CD_3CN for 1.5 h at room temperature.

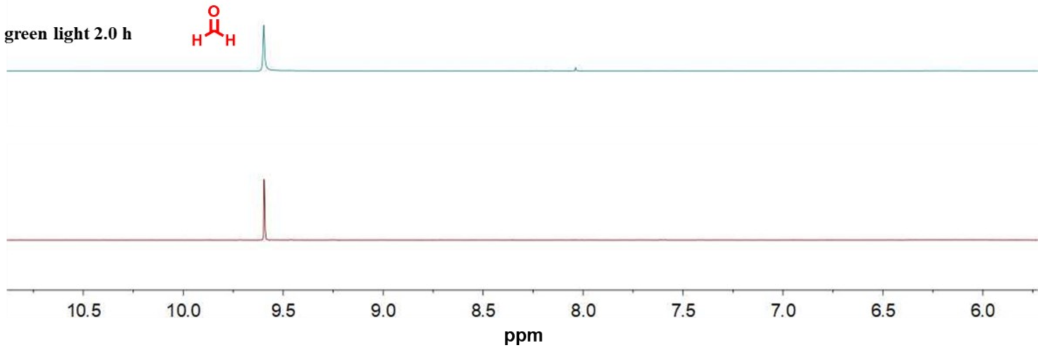

Figure S17. ^{1}H NMR spectrum of **HCHO** (2.0 × 10^{-2} mmol) in the presence of **G-I@Q[8]** (1.0 × 10^{-4} mmol) under green LED green irradiation in CD_3CN for 1.5 h at room temperature.

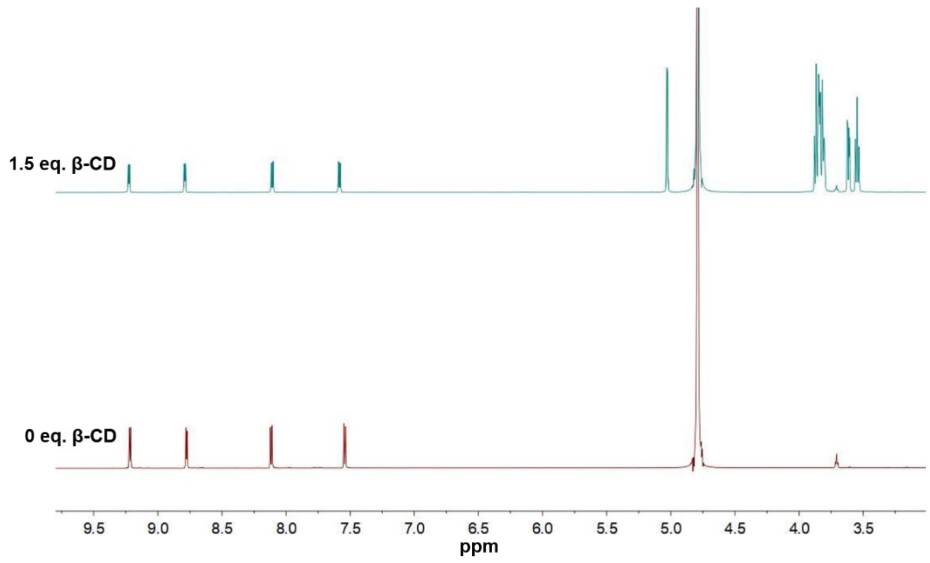

Figure S18. ^{1}H NMR spectra obtained for **G-I** (1.0 mM, D_2O) in the presence of 1.5 eq. of β-CD host.

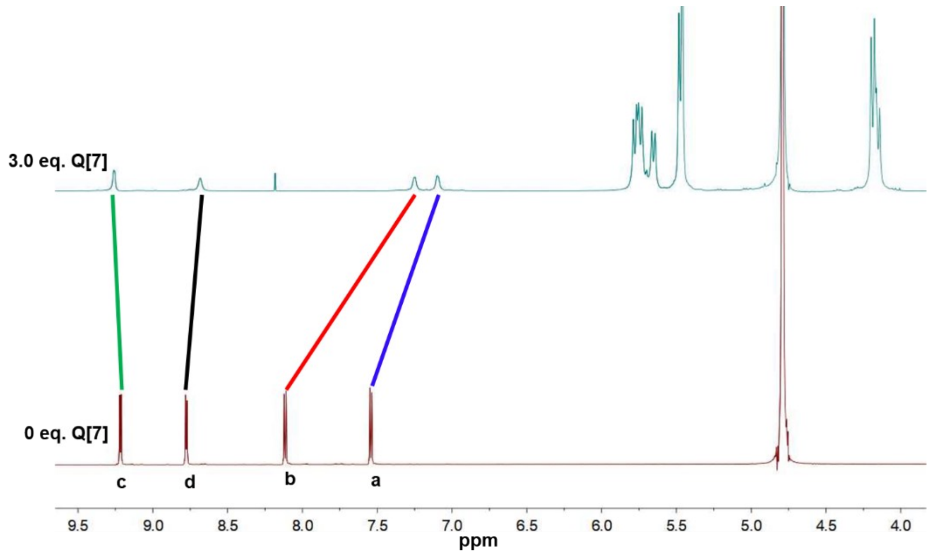

Figure S19. ^{1}H NMR spectra obtained for **G-I** (1.0 mM, D_2O) in the presence of 3.0 eq. of Q[7] host.

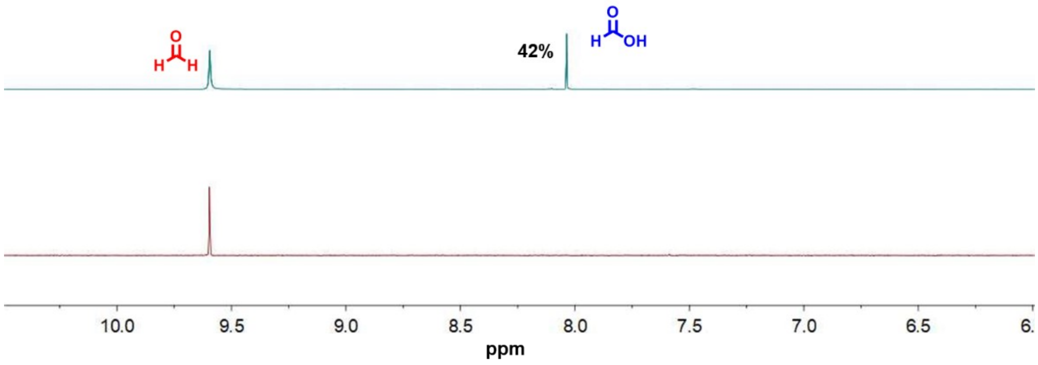

Figure S20. ^{1}H NMR spectrum of **HCHO** (2.0×10^{-2} mmol) in the presence of **G-I@Q[7]** (1.0×10^{-4} mmol) under blue LED light irradiation in CD_3CN for 1.5 h at room temperature.

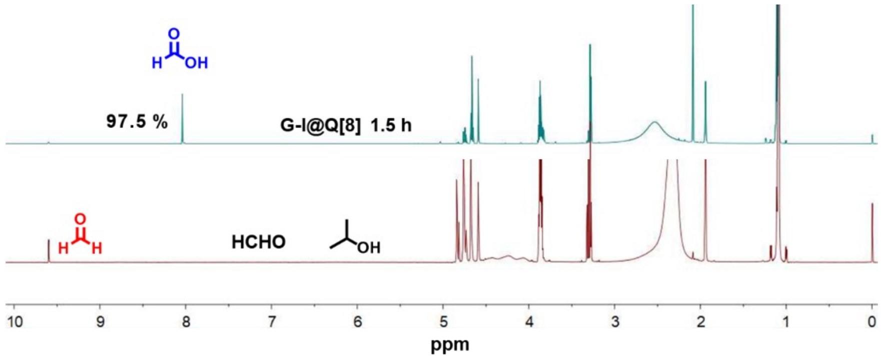

Figure S21. ^{1}H NMR spectrum of **HCHO** (2.0×10^{-2} mmol) in the presence of **G-I@Q[8]** (1.0×10^{-4} mmol) and Isopropanol (IPA) under blue LED light irradiation in CD_3CN for 1.5 h at room temperature.

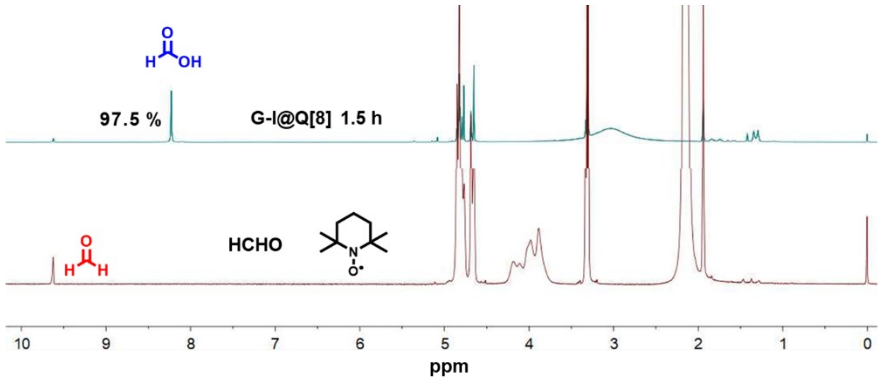

Figure S22. ^{1}H NMR spectrum of **HCHO** (2.0×10^{-2} mmol) in the presence of **G-I@Q[8]** (1.0×10^{-4} mmol) and 2,2,6,6-Tetramethylpiperidinooxy (TEMPO) under blue LED light irradiation in CD_3CN for 1.5 h at room temperature.

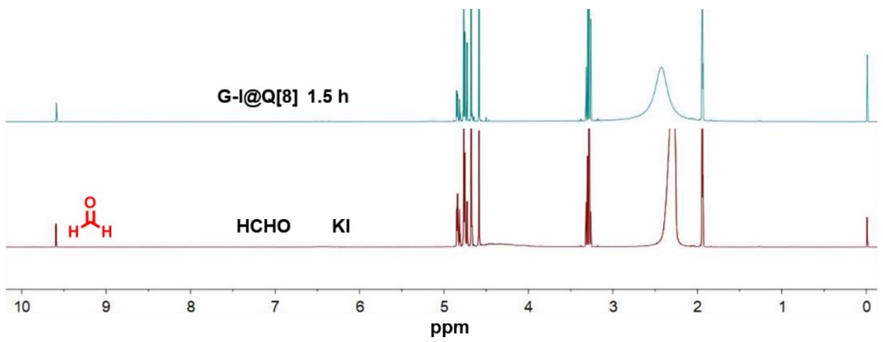

Figure S23. ^{1}H NMR spectrum of **HCHO** (2.0×10^{-2} mmol) in the presence of **G-I@Q[8]** (1.0×10^{-4} mmol) and KI under blue LED light irradiation in CD_3CN for 1.5 h at room temperature.

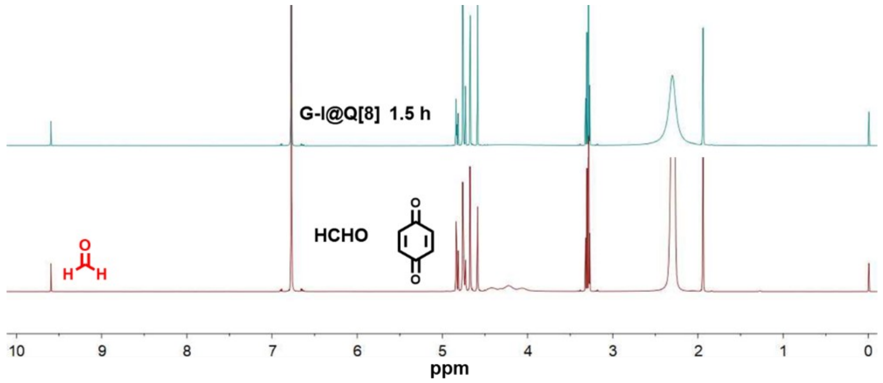

Figure S24. ^{1}H NMR spectrum of **HCHO** (2.0×10^{-2} mmol) in the presence of **G-I@Q[8]** (1.0×10^{-4} mmol) and 1,4-Benzoquinone (PBQ) under blue LED light irradiation in CD_3CN for 1.5 h at room temperature.

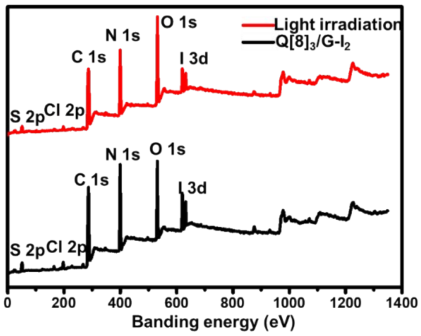

Figure S25. The full-scan X-ray photoelectron spectroscopy (XPS) spectra of C 1s, N 1s, I 3d, Cl 2p, S 2p and O 1s of **G-I@Q[8]** before (black) and after (red) light irradiation.

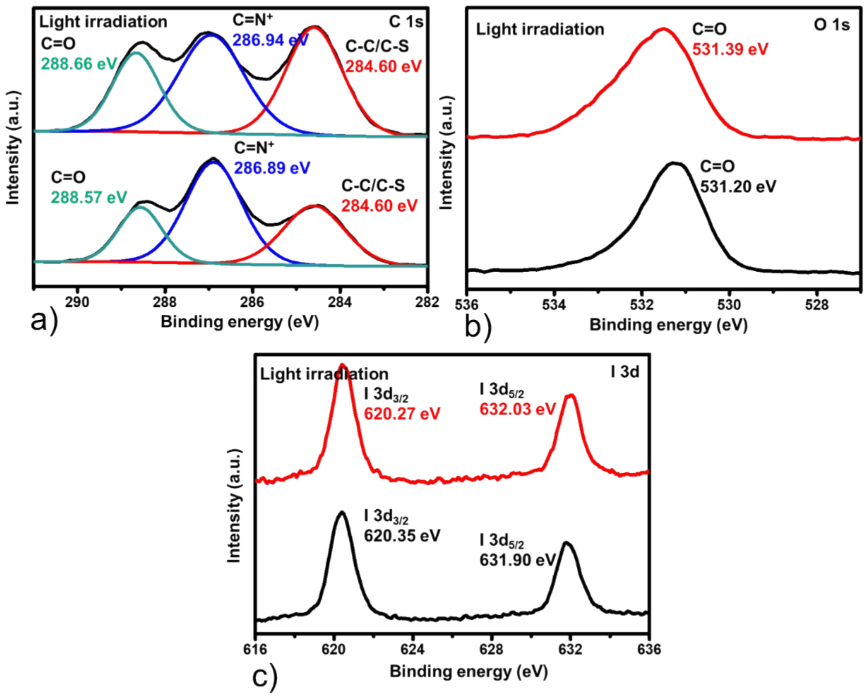

Figure S26. XPS spectra corresponding to a) C 1s, b) O 1s, and c) I 3d in the **G-I@Q[8]** systems before and after irradiation with light irradiation.

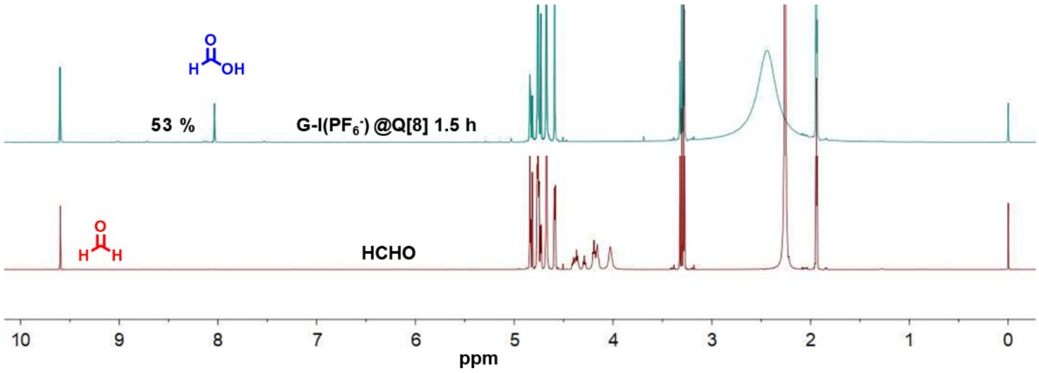

Figure S27. ^{1}H NMR spectrum of **HCHO** (2.0×10^{-2} mmol) in the presence of **G-I(PF_6^-)@Q[8]** (1.0×10^{-4} mmol) under blue LED light irradiation in CD_3CN for 1.5 h at room temperature.

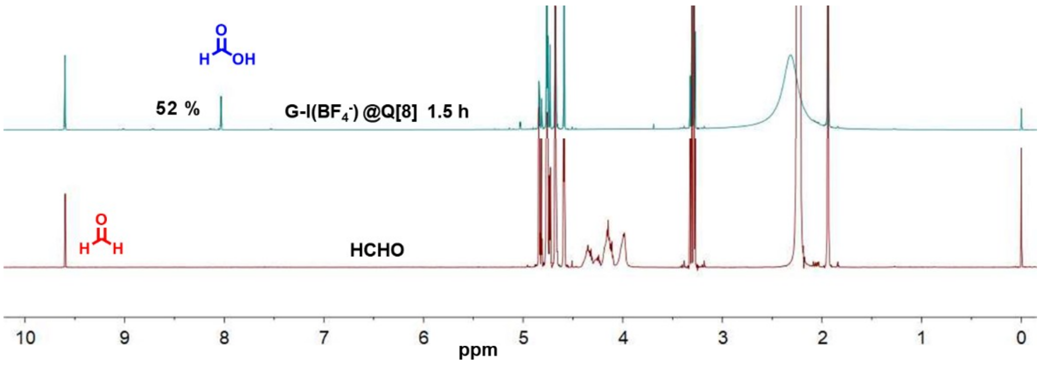

Figure S28. ^{1}H NMR spectrum of **HCHO** (2.0×10^{-2} mmol) in the presence of **G-I(BF_4^-)@Q[8]** (1.0×10^{-4} mmol) under blue LED light irradiation in CD_3CN for 1.5 h at room temperature.

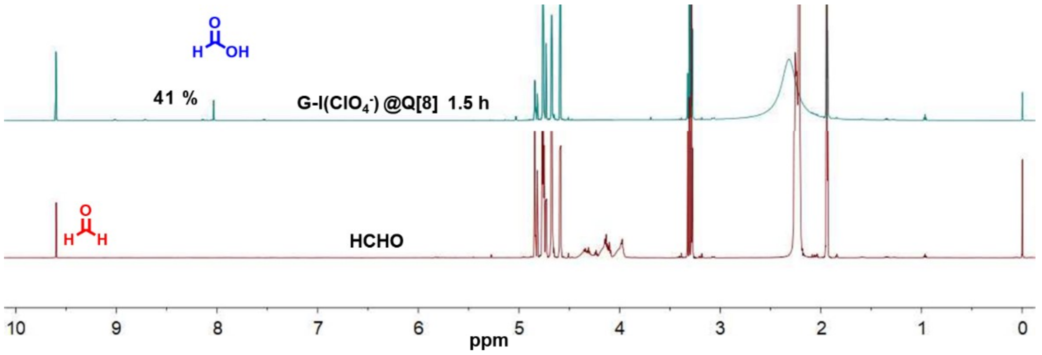

Figure S29. ^{1}H NMR spectrum of **HCHO** (2.0×10^{-2} mmol) in the presence of **G-I(ClO_4^-)@Q[8]** (1.0×10^{-4} mmol) under blue LED light irradiation in CD_3CN for 1.5 h at room temperature.

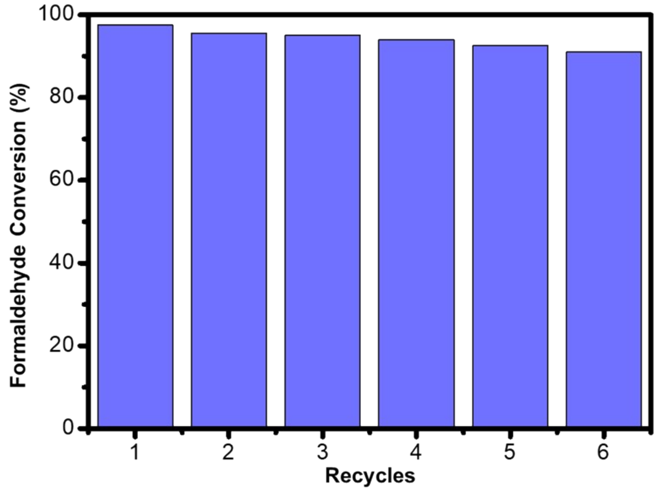

Figure S30. The formaldehyde degradation activity of G-I@Q[8] in six recycling tests under blue LED light source.

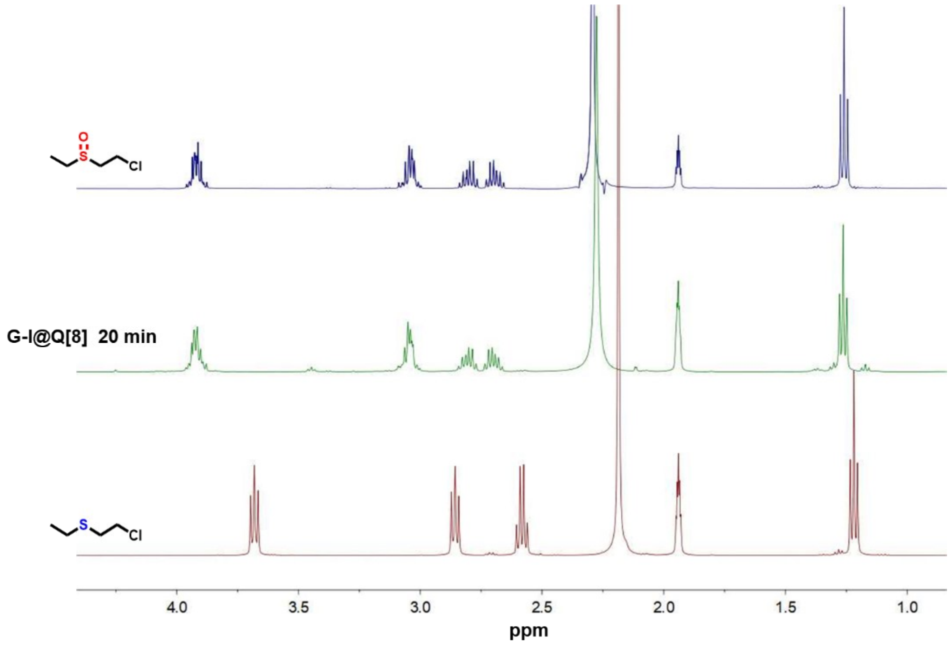

Figure S31. ^{1}H NMR spectrum of 2-chloroethyl ethyl sulfide (CEES) (2.0×10^{-2} mmol) in the presence of **G-I@Q[8]** (1.0×10^{-4} mmol) under blue LED light irradiation in CD_3CN for 20 min at room temperature.

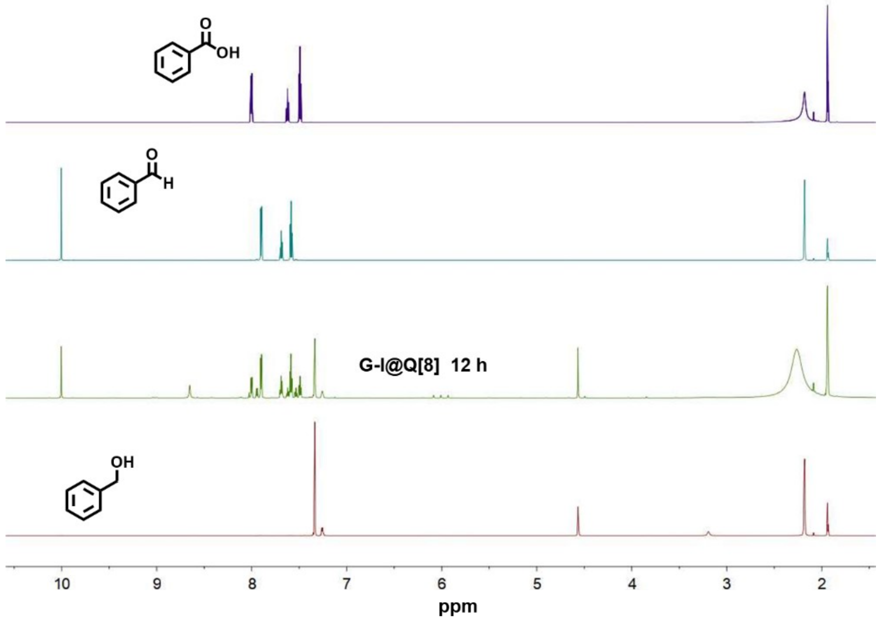

Figure 32. ^{1}H NMR spectrum of benzyl alcohol (2.0×10^{-2} mmol) in the presence of **G-I@Q[8]** (4.0×10^{-4} mmol) under blue LED light irradiation in CD_3CN for 12 h at room temperature.

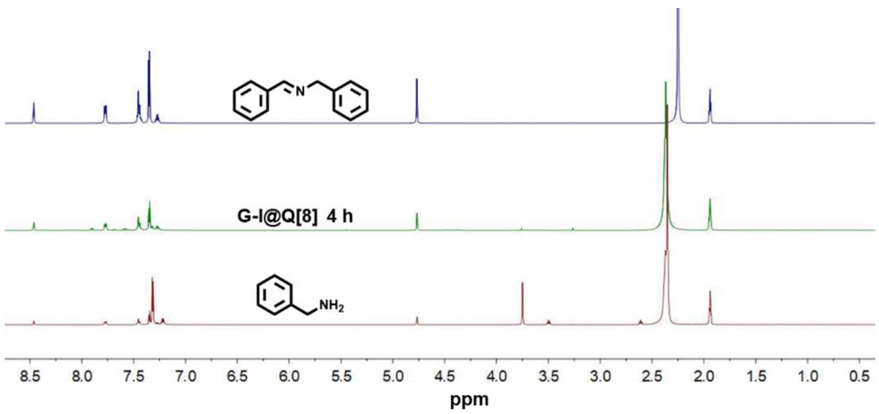

Figure S33. ^{1}H NMR spectrum of Benzylamine (2.0 × 10^{-2} mmol) in the presence of **G-I@Q[8]** (1.0 × 10^{-4} mmol) under blue LED light irradiation in CD_3CN for 4 h at room temperature.

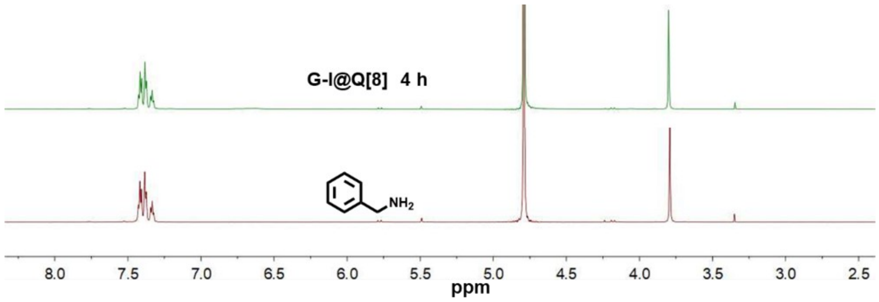

Figure S34. ^{1}H NMR spectrum of Benzylamine (4.0 × 10^{-2} M) in the presence of **G-I@Q[8]** (2.0 × 10^{-4} M for guest) under blue LED light irradiation in D_2O for 4 h at room temperature.

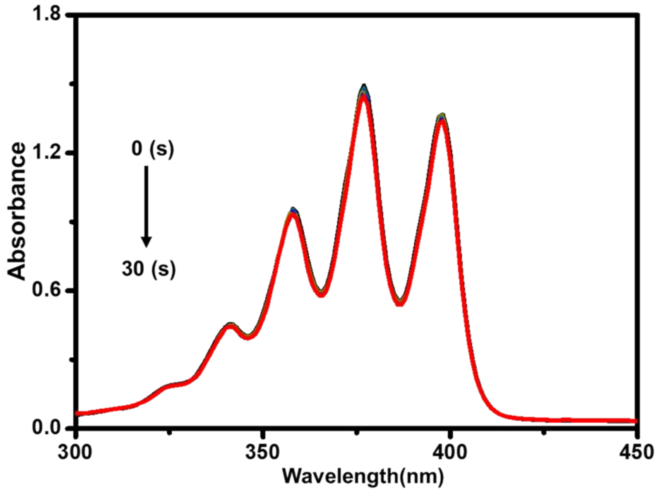

Figure S35. UV-vis spectral changes of ABDA (1.5 × 10^{-4} M) in the presence of **G-I@Q[8]** (4.5 × 10^{-6} mmol) under white light irradiation in CH_3CN at room temperature.

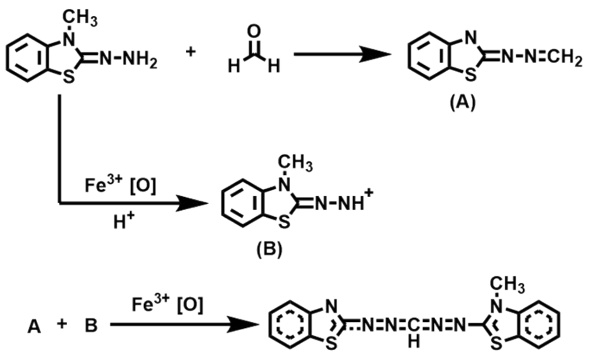

Figure S36. Flow chart of the chemical reaction between phenol reagent and formaldehyde.[2]

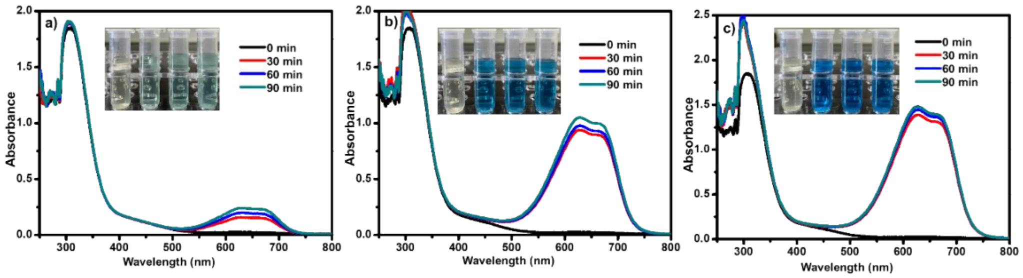

Figure S37. UV-Vis absorption spectra of a) the **G-I@Q[8]-cotton** fiber composite b) the **G-I-cotton** fiber composite and c) the untreated cotton fiber after 90 minutes of adsorption-degradation treatment (440−450 nm, 20W). And its time-dependent color figure after adsorption-degradation treatment.

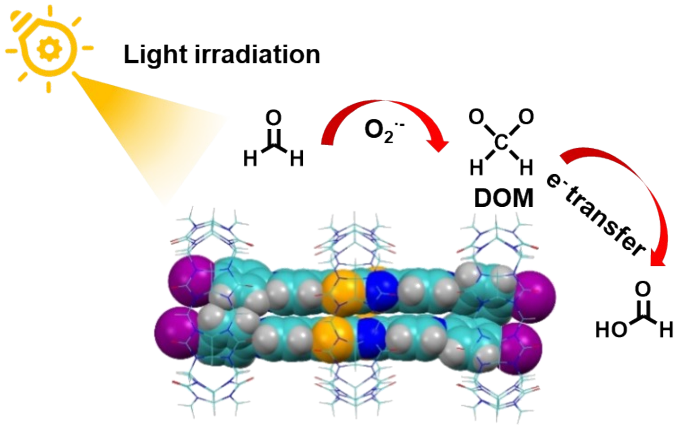

Figure S38. Photocatalytic oxidation mechanism of formaldehyde by **G-I@Q[8].**[4]

Table S1. Photophysical parameters of **G-I** and **G-I@Q[8]** at toom temperature.

	T(ns)	Φ(%)	k_r(μs^{-1})	k_{nr}(μs^{-1})
**G-I**	0.34	0.96	28.2	2912.9
**G-I@Q[8]**	2.85	0.42	1.5	349.4

Table S2. A Summary of Singlet Oxygen Quantum Yields of Recent Photosensitizers

Name	Photosensitizer	$^1O_2$ quantum yield	Reference
This work	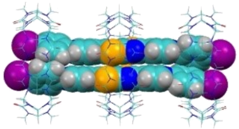	6.42	
Naph-α-TCy5	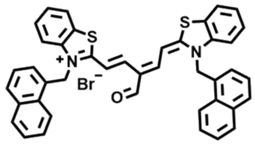	0.89	X. Zhang, * et al. *CCS Chem.* 2025, 7, 832-842
1.4Cl	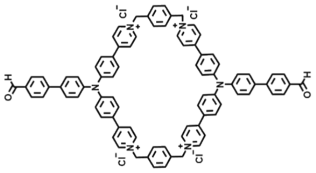	2.65	Y. Liu, * et al. *CCS Chem.* 2025, 10.31635/ccschem.025.202505567
Q[8]$_3$/G1$_2$	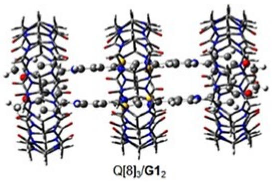 Q[8]$_3$/G1$_2$	3.94	X.-L. Ni, * et al. *Sci. China Chem.* 2024, **67**, 1605-1612.
BTBP-Q[8]	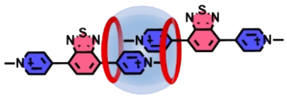	1.52	L.-B. Xing, * et al. *Langmuir* 2024, **40**, 19279−19286
APA2	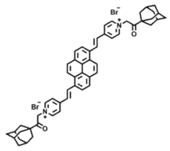	0.89	Y. Liu, * et al. *Biomacromolecules* 2022, **23**, 3549–3559

1·4Cl⁻	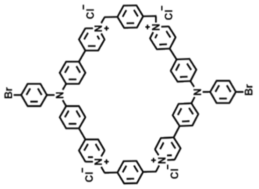	1.3	Y. Liu, * et al. Adv. Sci. 2022, **9**, 2201962
TCy5-Btz	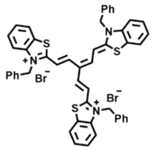	0.99	X. J. Peng, * et al. *Chem. Sci.*, 2021, **12**, 13809—13816
PTP	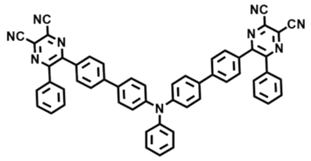	0.45	B. Z. Tang, * et al. Angew. Chem., Int. Ed. 2018, **57**, 15189–15193

The single crystal of **G-I@Q[8]** was obtained by evaporation of Q[8] (9.0 mg), G-I (2.6 mg) in a solution mixture (6.0 mL, HCl/H_2O, 1:1, v/v) within about several weeks. Crystal data and refinement for **G-I@Q[8]** was shown in Table S3.

Table S3. Crystal data and structure refinement for **G-I@Q[8]**.

Identification code	**G-I@Q[8]**
Empirical formula	$C_{98}\ H_{88}\ Cl_2\ I_2\ N_{52}\ O_{24}\ S_2$
Formula weight	2767.34
Temperature/K	100.00(10)
Crystal system	Monoclinic
Space group	P 1 21/n 1
a/Å	18.3550(4)
b/Å	27.2455(6)
c/Å	34.3754(7)
α/°	90
β/°	99.767(2)
γ/°	90
Volume/Å^3	16941.7(6)
Z	4
ρ_{calc}g/cm^3	1.085
F(000)	5617
Goodness-of-fit on F^2	1.040

Final R indices [I>=2σ (I)]	R_1 = 0.1041, wR_2 = 0.2814
Final R indices (all data)	R_1 = 0.1389, wR_2 = 0.3090
CCDC number	2473180

The single crystal of **G-I@Q[8]-HCHO** was obtained by immersing **G-I@Q[8]** complex crystals in a formaldehyde solution for three days. Crystal data and refinement for **G-I@Q[8]-HCHO** was shown in Table S4

Table S4. Crystal data and structure refinement for **G-I@Q[8]-HCHO**.

Identification code	**G-I@Q[8]-HCHO**
Empirical formula	$C_{100}\ H_{92}\ Cl\ I_2\ N_{52}\ O_{26}\ S_2$
Formula weight	2791.62
Temperature/K	100.0
Crystal system	Monoclinic
Space group	P 1 21/n 1
a/Å	18.2818(8)
b/Å	27.2752(10)
c/Å	34.8195(10)
α/°	90
β/°	101.584
γ/°	90
Volume/Å^3	17008.7(11)
Z	4
ρ_{calc}g/cm^3	1.090
F(000)	5676
Goodness-of-fit on F^2	0.953
Final R indices [I>=2σ (I)]	R_1 = 0.0776, wR_2 = 0.2069
R indices (all data)	R_1 = 0.1260, wR_2 = 0.2505
CCDC number	2473294

References:

1. J. Wang, L. Zhang, D. Zeng, W. Wang, R. Li, T. Jia, B. Cui, H. Chu and W. Wang, *Appl. Catal., B* 2023, **337**, 122983.
2. C. Huang, W. Dai, S. Deng, Y. Tian, X. Liu, J. Lin and H. Chen, *Chin. Chen. Lett.* 2024, **35**, 109429.

3. H. Nie, D. Hu, Z. Zeng, Y. Fan, Y. Zhai and X.-L. Ni, *Sci. China Chem.* 2024, **67**, 1605-1612.
4. Q. Liu, Y. Wang, M. Wen, Y. Guo, Y. Wei, G. Li, T. An, *Environ.Sci.-Nano* 2022, **9**, 4162–4176.

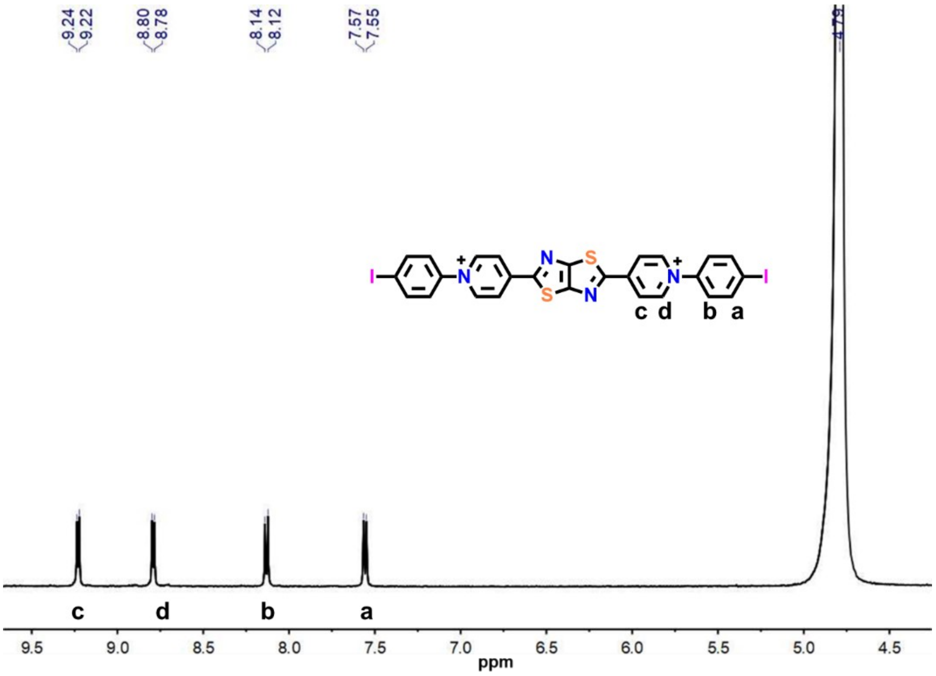

Figure 39. ^{1}H NMR spectra (500 MHz, D_2O, 298K) of **G-I**.

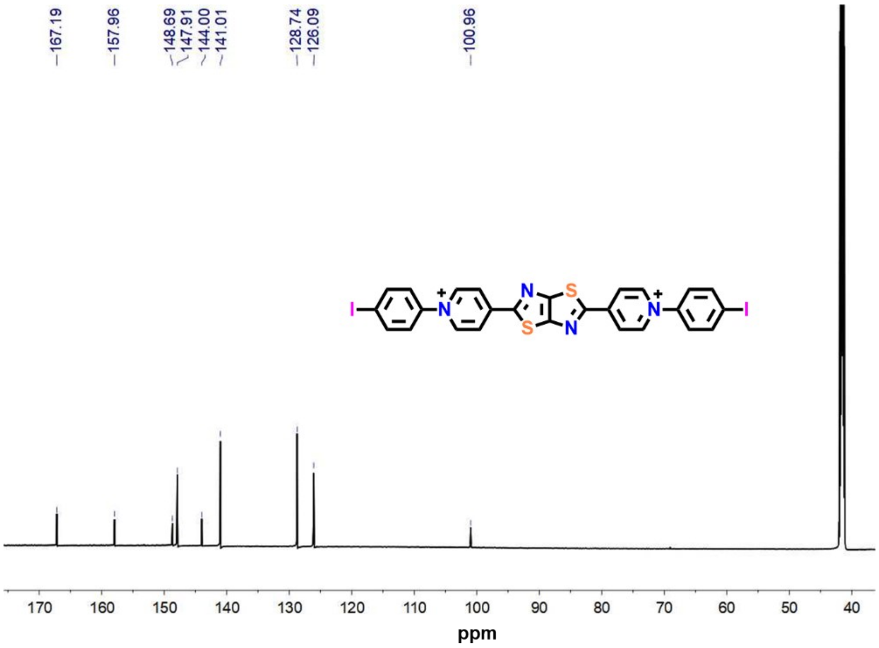

Figure S40. ^{13}C NMR spectra (176 MHz, DMSO, 298K) of **G-I**.
